# Where do you live and what do you do? Two questions that might impact your kidney health

**DOI:** 10.3389/fneph.2022.1011964

**Published:** 2022-10-05

**Authors:** Mabel Aoun, Dania Chelala

**Affiliations:** Faculty of Medicine, Saint-Joseph University, Beirut, Lebanon

**Keywords:** Social Determinants of Kidney Health, Altitude, Cold weather, Heat, Hypertension, Occupation, High-income and low- and middle-income countries, Seasonality

## Abstract

In many cases the social determinants of health need to be assessed through their interaction with environmental factors. This review looks at the impact of physical location and occupation of individuals on their kidney health. It examines the effect of living at high altitude on kidney function and the relationship between extreme cold or hot temperatures and the incidence of kidney injury. It reviews as well the many occupations that have been linked to kidney disease in high-income and low-and-middle-income countries. As a conclusion, this overview proposes preventive recommendations that could be individualized based on weather, altitude, socio-economic level of the country and occupation of the individual.

## Introduction

In 2005, the World Health Organization (WHO) launched the commission on Social Determinants of Health with the aim to address the impact of social hazards on health and health inequities (1). Progressively, the global nephrology community started to show interest in social determinants of kidney health which have been assembled under five categories: first the economic stability including employment, income and food, second education, third health and healthcare access, fourth social, community and context, the fifth category being neighborhood and built environment (2). In fact, social determinants of health and their impact on health and disease are better analyzed through their interaction with other domains that influence individual health. Therefore, a multisectoral approach has been suggested to treat the complexity of the fundamental social, environmental, political and economic causes of kidney health. This paper aims to review the impact of physical location and occupation of individuals on their kidney health.

The first section of this review analyzes the physical environment that may affect kidney health with a focus on altitude, weather and other atmospheric variables such as humidity. There is evidence that the incidence, prevalence, level of care and outcomes of chronic kidney disease (CKD) patients are variable according to the geographical location (3). This review will look at the kidney health at high altitude in the world regions that include the highest summits. On the other hand, it will examine the evidence beyond the association between cold temperatures, the increase in blood pressure levels and whether this could indirectly affect the kidney function. It aims also to overview climate change and global warming as causes for excessive heat, dehydration and acute kidney injury. Finally, it gives a brief about other atmospheric variables that may impact kidney health like air pollution and humidity and their role in the exacerbation of some kidney diseases such as lupus nephritis flares.

The second section reviews the evidence behind the impact of occupation on kidney health of workers. In this part, occupational kidney disease is analyzed based on five main socio-economic, temporal and/or geographical subsections. First, it summarizes the old occupational nephrotoxic hazards, heavy metals, infections and pesticides that were well-known before the first report of El Salvador in 2002. Second, it reviews the occupational kidney disease or CKD of unknown etiology (CKDu) in Sri Lanka among agricultural workers exposed to contaminated water. Third, it looks at the evidence of heat stress in the Mesoamerican nephropathy among sugarcane workers. Fourth it overviews the types of occupational kidney disease reported in low- and middle-income countries (LMICs) other than Sri Lanka and Central America. Finally, it reviews the different causes of occupational kidney disease in high-income countries.

## Search strategy

### “Altitude and kidney health”

A search for articles was performed in PubMed by combining the following terms under “Title/Abstract”: “Kidney OR Renal” AND “Altitude” NOT “ALTITUDE Trial”. The filter human was added and this search led to 271 abstracts that were reviewed and 40 articles were selected.

### “Seasonal variation and the kidney”

A search for articles was performed in PubMed by combining the following terms under “Title/Abstract”: “Kidney OR Renal” AND “Seasonal variation OR Extreme temperatures”. The filter “humans” was added. Seventeen out of 120 abstracts were selected and papers reviewed.

### “Cold and the kidney”

A search for articles was performed in PubMed by combining the following terms under “Title/Abstract”: “Kidney OR Renal” AND “Cold weather OR Cold temperature” NOT “Transplant OR Transplantation”. The filter “humans” was added. This search led to 19 abstracts and 11 papers were selected and reviewed.

### “Heat and the kidney”

A search for articles was performed in PubMed by combining the following terms under “Title/Abstract”: “Kidney OR Renal” AND “Heat OR hot temperature” NOT “Heat stroke” NOT “Heat shock protein OR Heat-shock protein”. 36 out of 198 articles were selected and reviewed.

### “Occupation and the kidney”

A search for articles was performed in PubMed by combining the following terms under “Title/Abstract”: “Kidney OR Renal” AND “Occupation OR Occupational OR Job” NOT “Cancer” NOT “Stone OR Lithiasis”. Filters added to this search were “humans” and timeline “1990 until July 2022”. This search led to 1388 results. These studies’ abstracts were reviewed to extract all types of clinical studies that described or analyzed occupational kidney diseases. Studies that covered only the kidney toxicity of heavy metals including lead, cadmium, arsenic, mercury and uranium were not included in the final tables but the most recent and relevant were discussed in the text. Among the 87 studies reporting infectious diseases as an occupational risk factor for kidney disease, thirty studies about hantavirus, schistosomiasis, brucellosis, malaria or dengue were not added to the tables. A total of 57 studies on leptospirosis were included because it was the most frequently reported infectious disease associated with occupational renal failure. Finally, 968 abstracts were reviewed, reviews and systematic reviews were not included in the final tables but summarized in the text. A total of 150 articles about occupational risk factors for kidney health were finally selected and divided into those conducted in low and middle-income countries (100 studies) or in high-income countries (50 studies). Data extracted from these articles included the study design, number of participants, occupation, occupational exposure, health outcomes and kidney outcomes.

## Altitude and the kidney

### Distribution of populations at high altitude

Living at high altitude has been defined as living at ≥2500 meters (m) above sea level. It is globally estimated that 81.6 million at ≥2500 m (4). The highest summit in the world is Mount Everest in Nepal located at 8848 m in the Himalayas’ mountains. Other summits exist in Asia, located in India and Indonesia. In Africa, the Eastern African mountains are located in Tanzania with Kilimanjaro being the highest summit of 5895 m, extending along Tanzania’s northern borders with Kenya. Also in Africa, the highest peak in Ethiopia is Ras Dejen (4533 m). In Europe, the Alps’ highest summit is Mont Blanc (4809 m) between France and Italy. Running along the western edge of South America, we find the Andean mountains with the highest summit in Chile at 6140 m. The Andes extend over seven countries: Argentina, Chile, Bolivia, Peru, Ecuador, Colombia and Venezuela. Ethiopia has the highest absolute number of inhabitants living at ≥1,500 m and ≥2,500 m, China has the highest at ≥3,500 m (4).

High altitude induces physiological responses in the human body and adaptation of several organs to hypoxia which make it an important public health issue.

### Kidney’s physiological response to high altitude

**Figure 1:**
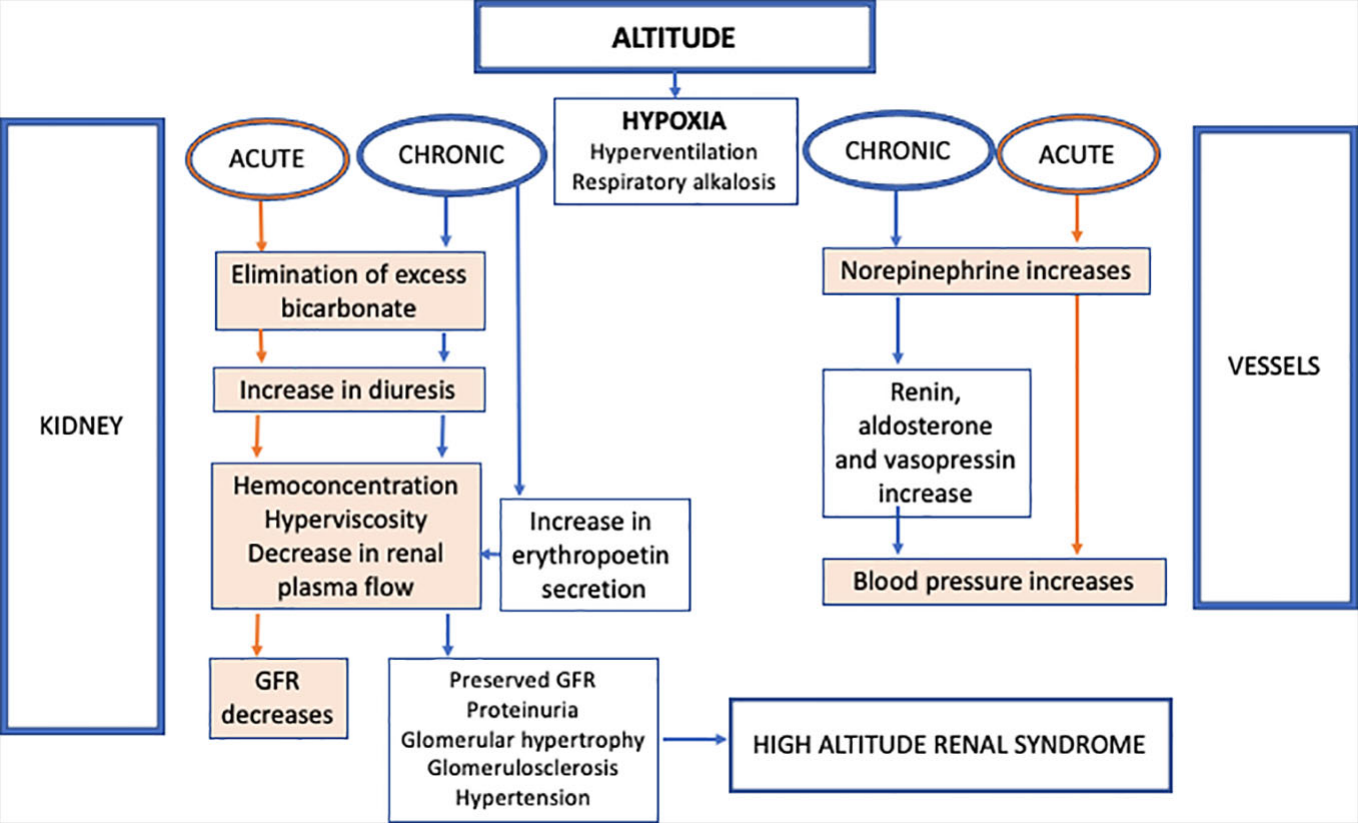
Effect of altitude on kidney function and blood pressure.

A summary of the effects of altitude on kidney function is illustrated in **Figure 1**. One of the first experimental studies that analyzed the cause of albuminuria at high altitude was published in 1987 and it showed that albuminuria after acclimatization at 4846 m is due to increased glomerular capillary permeability (5). A systematic review by Palubiski et al. studied the kidney physiological response to high altitude and highlighted the integrated response between the kidney, the lungs and the cardiovascular system when adapting to hypoxia at high altitude (6). They differentiated between acute and chronic kidney response to high altitude and hypoxia. The acute response starts with hyperventilation, leading to respiratory alkalosis; this latter is compensated by the role of the kidneys in eliminating the excess of bicarbonate (6, 7). There is an increase in diuresis and hemoconcentration that return to baseline at 2100m. This seems to precede the increase in erythropoietin secretion. In chronic high-altitude states > 2100 m, the erythropoietin increases and this is the most solid evidence revealed by the literature; however, there are no studies at altitudes lower than 2100 m (6). When looking at the glomerular filtration rate (GFR), an acute exposure to altitude is associated with a decrease in GFR, which is secondary to the decrease in renal plasma flow which in turn is due to hemoconcentration and hyperviscosity. When the exposure to altitude is chronic, GFR returns to a normal level despite the persistence of a diminished renal plasma flow probably due to sympathetic nerve hyperactivity starting at day 6. This leads to an increase in post-glomerular resistance and an increase in the filtration fraction (6, 8–10). The details of the mechanism that maintain GFR at chronic high-altitude exposure despite the tissue hypoxia, the hyperviscosity, the erythropoietin secretion and the affected blood flow are still unclear. It is also uncertain if the increase in blood pressure is omnipresent at high altitude, if it is dependent on high erythropoietin levels or not and if hypertension affects the GFR. The increase in blood pressure seems to be related in the first days to an acute secretion in norepinephrine. Later, the effects of renin, aldosterone and vasopressin on top of norepinephrine contribute to explain the mechanism of hypertension. Finally intrarenal tissue hypoxia seems to be reversible after the individual returns to lower altitude (6).

The high-altitude renal syndrome is a term that includes all the renal manifestations after chronic exposure to high altitude. Historically, three summits in the world are among the eldest places where humans have lived at > 2400 m: the Ethiopian summit in the North African highlands, the Andes mountains in South America and the Tibet in the Himalayan mountains in Asia (11). The common kidney manifestations in the three regions following high altitude are polycythemia (increase in erythropoietin secretion), hyperuricemia, elevated blood pressure, decreased renal blood flow with preserved GFR, glomerulomegaly and albuminuria. Despite their genetic adaptation to high altitude, the residents of these three regions have different characteristics. Compared to Andeans, the Tibetans historically had the longest exposure to high altitude thus they less erythrocytosis and less chronic mountain sickness. The Andeans have low birth weight newborns because of the hypoxia but to a lesser degree than some Europeans living at high altitude for shorter historical periods of time. In addition, some parameters of adaptation are still underreported in the Ethiopians like the prevalence of the chronic mountain sickness or Monge’s disease. In fact, the chronic mountain sickness does not increase with increasing altitude but it is rather linked to shorter adaptation and also to heavy metal toxicity like cobalt and nickel that induce an increase in erythropoietin and are found in the mining communities in Chile (2800 m) (11).

### Clinical studies across the world that reported the effect of high altitude on kidney health

**Figure 2:**
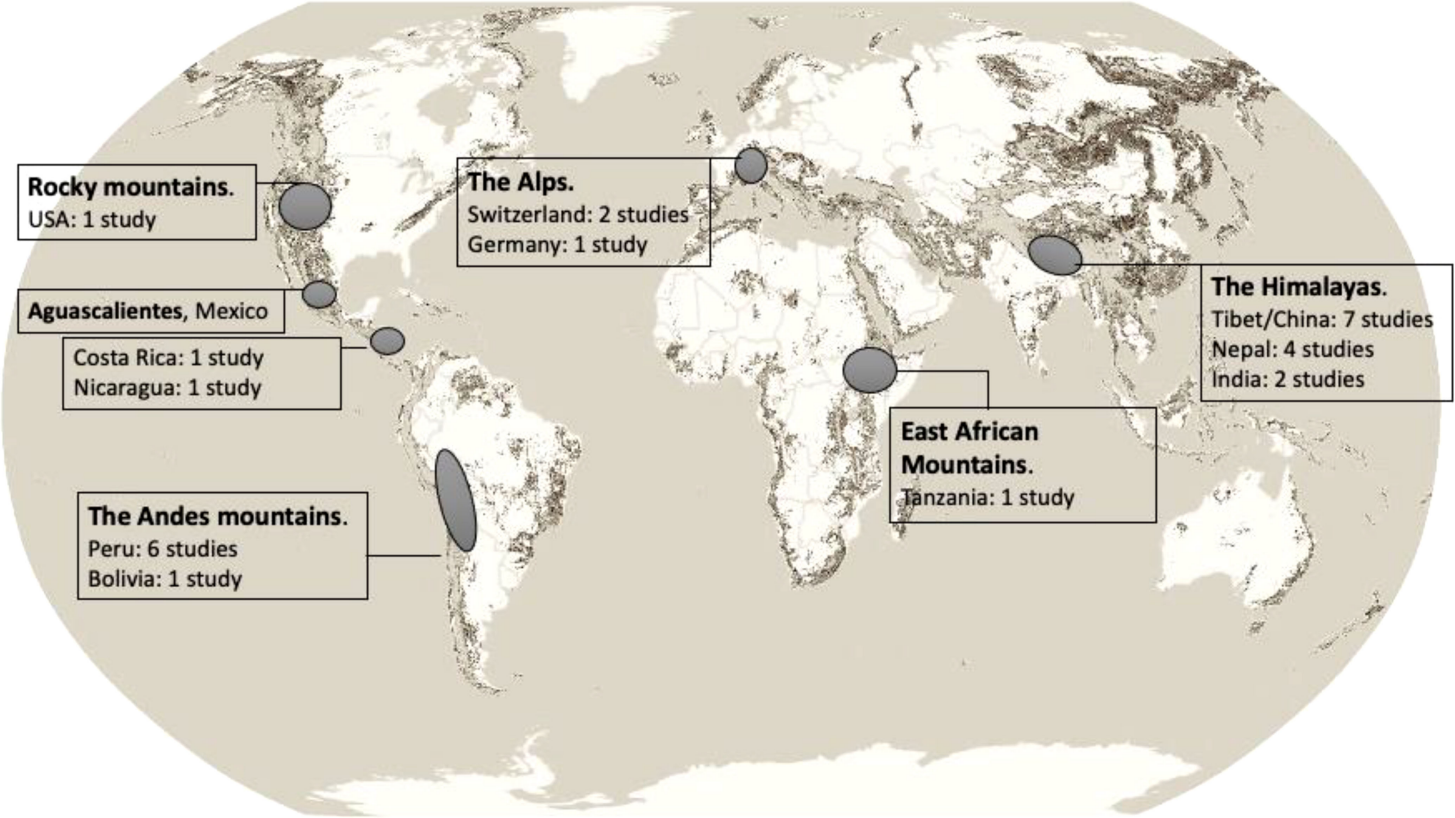
Distribution of studies about high altitude and the kidney across the globe.

The impact of high altitude on the kidney has been studied since the 70s with one of the first interventional trials done on 7 climbers in East African mountains (12). They studied the kidney response and acclimatization in climbers who developed transient proteinuria. During the last four decades, most of the clinical studies come from Tibet, Nepal, Peru, China and India (5, 12–40). They are summarized in **Table 1** and **Figure 2**. Several case reports described as well the kidney injury after a rapid ascent to very high altitudes. One of the cases comes from China in a previously healthy man who was suddenly exposed to an altitude of 5200 m and developed AKI (16). Another case of a 30-year-old healthy man who climbed the Everest at 5300 m in 11 days was reported with hypertensive crisis and reversible acute kidney injury (18).

**Table 1:** Clinical observational and interventional studies about altitude and the kidney.

Reference	Type of study	Number of participants	Altitude in meters	Country	Outcome
***Studies from Africa***					
Pines A. 1978 (12)	Interventional with lab test (Proteinuria)	7 climbers during 6 weeks	5890 m	East African mountains (Tanzania)	High altitude acclimatization and illness. Proteinuria
***Studies from Asia***					
Winterborn MH, 1987 (5)	Experimental		4846 m		High altitude acclimatization and albuminuria
Singh MV, 2003 (13)	Clinical trial studying blood gases and blood viscosity and renal blood flow	15 male sea-level volunteers	Prolonged mountain sojourns at 3500 and 5800 m	India, from Delhi sea-level to 3500 m for 60 days then 5800 m for 70 days and 7 days after return to sea level	The kidney seems to protect the body against hypoxia and plays a major role in acclimatization: pH significantly increased at 3500 and 5800 m; There was 38% decrease in pO2 and renal plasma flow at 5800 m. Blood viscosity increased and was totally reversible after return to sea level.
Cumbo TA, 2005 (14)	Clinical trial with collection of venous blood to measure bicarbonate	52 lowland-dwelling persons	4250 m	Nepal After they completed a religious pilgrimage in the Nepal Himalayas	Low oxygen saturation levels were associated with higher bicarbonate levels but not with acute mountain sickness
Chen W, 2011 (15)	Clinical trial	1289 Tibetans were tested for hematuria, albuminuria and eGFR	>3658 m Lhasa; 4200 m Dangxiong County	Tibet (four districts of Lhasa and 8 villages from Dangxiong County	High prevalence of low eGFR (2.1%), hypertension and albuminuria (16.2%). Albuminuria was strongly associated with hyperuricemia and high hematocrit.
Yijiang Z, 2013 (16)	Case report	Healthy young man	5200 m	China	Acute kidney injury
Suh KS, 2015 (17)	Clinical trial that compared mountain climbers: 10 transplant and donors to 6 healthy subjects	10 subjects: Athletic transplant donors and recipients, moslty liver and one kidney recipient and his donor	Reached 5150-6189 m of mountain climbing	Island Peak, Himalayas, Nepal	Kidney transplant Blood levels of immunosuppressants were well maintained; erythropoietin levels reached higher levels in patients treated with tacrolimus
Gilbert-Kawai E, 2016 (18)	Case report	Healthy young man	5300 m	Mount Everest	Hypertensive crisis and Acute kidney injury
Zhang L, 2018 (19)	Cross-sectional study between 2014 and 2016	1707 subjects >=35 years, blood and urine samples were tested	Three levels	Tibet: Linzhi (2900 m), Lhasa (3650 m) and Anduo (4700 m)	Prevalence of CKD was high at all levels and higher than the known worldwide prevalence rate varying between 18.3% in men living in Lhasa and ~37% in women living in Linzhi and Anduo
Phelan B, 2019 (20)	Case report	A climber with a kidney transplant		Nepal	
Zhao, 2020 (21)	Retrospective study comparing Han Chinese and native Tibetans	369 patients with type 2 diabetes and biopsy confirmed diabetic nephropathy	> 2000 m	China	Living at high altitude > 2000 m was independently associated with progression to ESKD in Han Chinese patients with diabetic nephropathy, but not native Tibetans
Fitria L, 2020 (22)	Cross-sectional studying urine and blood tests and DEGREE questionnaire	354 healthy male rice farmers	Sea level Karawang against 450 m Bogor	West Java, Indonesia	Prevalence of CKDu in all sample is high 24.9%. Patients in Bogor (higher altitude) had higher risk of CKDu, a result that is not aligned with results from El Salvador or India
Wei H, 2021 (23)	Retrospective study between 2014 and 2021	190 Henoch Schonlein Purpura (HSP) patients	Three levels of altitude compared (<2700 m, 2700-4000 m and > 4000 m)	Tibet	Henoch-Schonlein purpura patients had more severe digestive complications at the highest altitude >4000 m (4.8% of patients) and higher risk of kidney injury
Li X, 2021 (24)	Retrospective study in a single center between 2015 and 2018	6512 pregnant women	Average of 3300 m (1504-5545 m)	Tibet	Pregnancy-related AKI: based on KDIGO, 2.09% had AKI; and high altitude was found to be associated with more adverse fetal outcomes like low birth weight and intrauterine growth restriction especially in those with uncontrolled blood pressure
Hamilton SA, 2022 (25)	Cross-sectional study of association between eGFR and heat, low altitude	Random sample of 11,119 subjects from rural and urban locations in Northern and Southern India	391 m	India (India has part of Himalaya, as well as Bhutan, China and Nepal)	No association of CKDu with low altitude or temperature; risk factors were male, older age and living next to cropland
Wang H, 2022 (26)	Retrospective study of kidney biopsies of patients with high-altitude polycythemia (HAPC) between 2016 and 2020	416 biopsies in 190 females and 210 males from native inhabitants: 17 had HAPC-related kidney disease	>2500 m for > 10 years	Tibet is an autonomous region of China sharing Mount Everest with Nepal (Tibetan plateau (4782 meters) on the northern side of the Himalayas). Capital Lhasa.	Median Proteinuria=2.5g, 9 Hypertensive, 3 out of 17 GFR<60 mL/min The histopathological findings revealed glomerular hypertrophy, GBM thickening and features of glomerulosclerosis and vascular remodeling, hyalinosis and fibrosis.
***Studies from Europe and USA***					
Ghahramani N, 2011 (27)	Retrospective study	Data from the National Health and Nutrition Examination Survey III (NHANES III) to examine the association between altitude of residence and eGFR. Data from the United States Renal Data System (USRDS) to study the association between altitude and prevalence of ESRD.	Different levels compared: All below 3695 feet or 1126 m	USA	eGFR increased at higher altitudes. Prevalence of ESRD decreased at higher altitudes.
Königsrainer I, 2012 (28)	Clinical trial	10 male kidney transplant recipients, Cycling against 10 healthy controls	Ascending >1800 m above sea level	Germany (Alps)	The immune response genes were over-represented after exhaustive exercise in healthy controls but not in transplant patients on immunosuppression. Question: should these patients reduce their immunosuppressants before exhaustive exercise.
Cippà PE, 2016 (29)	Retrospective study of kidney biopsies between 2000 and 2010	477 kidney transplant patients	Three levels of residence: <400, 400-600, >600 m)	Switzerland	Significantly higher rates of hemoglobin and arteriolar hyalinosis in patients living at higher altitude
Biollaz J, 2021 (30)	Clinical trial	18 healthy male resting volunteers flown by helicopter to an altitude of 4559 m	4559 m within 44 hours	Switzerland, Lausanne, Regina Margherita Hut	Kidney function was not affected by altitude; there was minor albuminuria. Renal dysfunction and water retention are not causes of acute mountain sickness (AMS) (no difference in kidney function and water retention between those who developed or not AMS)
***Studies from Mexico, Central and South America***					
Perico N, 2005 (31)	The Project for Renal Diseases in Bolivia started in 1992, large screening for kidney disease	Prospective randomized study in 26 patients of mixed Indian and European origin evaluating patients with altitude polycythemia and proteinuria>150 mg/d	Born at altitudes of 3200 to 4000 m, who had lived in La Paz (3600 m) for at least 1 year	Bolivia	Enalapril reduced proteinuria in the treated arm and protected their kidney
O’Donnell JK, 2011 (32)	Clinical trial	771 individuals	Max 2106 m	Nicaragua	Low altitude and CKDu
Gonzales GF, 2015 (33)	Clinical trial that evaluated blood levels of several markers including those of kidney function	487 subjects	Living at 4100 m	Peru	High uric acid, high hemoglobin and obesity was associated with kidney disease
Harhay MN, 2016 (34)	Costa Rican Longevity and Health Aging Study (CRELES)	2657 adults born before 1946. Analysis of risk factors associated with eGFR< 60.	Levels around 1000 m	Costa Rica	Adjusted odds ratio of CKD =1.28 with every 200 m increase in altitude
Hurtado-Arestegui A, 2018 (35)	Cross-sectional study	293 Healthy subjects: 168 living at high altitude and 125 at sea level	3640 m	Peru	Dwellers at high altitude have more proteinuria, higher hemoglobin and lower eGFR
Carrillo Larco RM, 2019 (36)	Cross-sectional study	4208 people	Lima sea level compared to regions (Puno) at 3825 m	Peru	Higher altitude and level of urbanization were associated with worse kidney function
Bravo-Jaimes K, 2020 (37)	Retrospective study	720 hemodialysis patients including 163 that lived > 2000 m above sea level	>2000 m	Peru	Mortality was not different
Gutierrez-Peña M, 2021 (38)	Retrospective study	2827 patients with ESKD between 2012 and 2020	Up to 3050 m	Aguascalientes Mexico, of an average of 19°C of year-round temperature	A high rate of prevalence of ESKD and > 50% CKDu with a peak in males between 20 and 40 years; predominance of focal segmental glomerulosclerosis on biopsies. Altitude was not analyzed but could be one hypothesis
Steele AR, 2021 (39)	Clinical trial that estimated renin, aldosterone, kidney function	14 Andean highlanders, males with CMS vs 10 without CMS	Andean Mountains	Peru	Negative correlation between plasma renin activity and GFR in both groups
Vizcarra-Vizcarra CA, 2022 (40)	Case report	Peritoneal dialysis patient		Peru	High-altitude pulmonary edema

### The effect of altitude on people with established chronic kidney disease

People living at high altitude might have chronic kidney disease (CKD) similarly to the general population. However, there is paucity of data about the effect and consequences of high altitude on the progression of chronic kidney disease and on patients on dialysis or kidney transplant recipients. Luks et al. discussed the possibility of kidney function deterioration secondary to systemic hypoxemia at high altitude and the risk of acute motion sickness that CKD patients might experience (41). Because CKD patients have insufficient production of erythropoietin, they develop less increase in hemoglobin secondary to hypoxia at high altitude, this would decrease their oxygen delivery to tissues and induce dyspnea (41). Metabolic acidosis of chronic kidney disease might protect patients against the acute mountain sickness and the alkalosis induced by high altitude (41). Hemodialysis and peritoneal dialysis patients might be at a higher risk of volume overload when exposed to high altitude (40, 42). Bravo-Jaimes et al. showed that the mortality of hemodialysis patients in Peru was not different whether they lived at low or high altitude (>2000 m) (37). Studies from Europe and Nepal showed that kidney transplant recipients have higher hemoglobin levels in these patients when exposed to high altitude especially when treated with tacrolimus. They also have a higher level of arteriolar hyalinosis on biopsies, a less immunological protective reaction to physical exercise but stability of immunosuppressant levels (17, 20, 28, 29).

### Kidney health and the occupation at altitude in the space

People working at high altitude in the space like flight crews or frequent flying passengers are increasing in number. This exposure to high altitude can impact their health because of the physiological changes of the body secondary to the decrease in atmospheric pressure. Moreover, there is a decrease in the partial pressure of oxygen which might lead to acute hypobaric hypoxia (43). This state will cause a redistribution of the blood to the main organs, the heart and the brain, inducing tachycardia and a decrease of the kidney perfusion as well as peripheral cyanosis (43). This is different from another entity called decompression sickness which is secondary to the formation of nitrogen bubbles in tissues and blood when exposed to an acute decrease in environmental pressure (43). Nitrogen accumulation leads to tissue ischemia, dyspnea, chest pain and sometimes to cardiac arrest.

### Drug intake in patients with CKD at high altitude

Luks et al. made some suggestions regarding medications’ intake in CKD patients when exposed to short journeys at high altitude. There is no evidence of the need to change any chronic medication in these patients (41). Because of the risk of fluid retention in the context of acute motion sickness, patients susceptible to fluid retention should monitor their weight and adapt their diuretics (41). Chronic antihypertensive medications that inhibit the renin-angiotensin system are preferred over dihydropyridine calcium channel blockers because of the highest risk of proteinuria at high altitude. In this group of patients, anemia of these patients at high altitude seems to be corrected with lower doses of erythropoietin compared to patients at sea level but they have higher risk of hypertension and thrombotic events (41). Finally, medications used for acute mountain sickness need to be adjusted according to the kidney function. The best way to avoid mountain sickness for chronic kidney disease patients remains slow ascent and descent.

## The weather and the kidney

### Weather and climate

Weather and climate both describe atmospheric conditions like temperature, air pressure, precipitation, wind and humidity. Weather refers to the short-term atmospheric conditions or specific daily state of the atmosphere. Climate is the average of weather conditions of a region, throughout the year, sometimes averaged over a series of years, 30 years or more. The climate is classified as tropical, dry, polar, temperate or continental. The climate change globally is leading to a composite of warmer temperatures even in cold climates. This doesn’t mean that snow and extreme cold temperatures will become mild, on the opposite higher temperatures might lead to more precipitations and storms. This is why it is very important to study extreme temperatures and their impact on health.

### Seasonal variation and the kidney

Seasonality is an important emerging factor that has been globally addressed. Several studies have assessed the effect of seasonality on the variation in blood pressure, kidney function, cardiovascular outcomes and/or laboratory biomarkers. Although cold exposure has not been extensively studied as heat exposure, several reports demonstrated the deleterious impact of very cold temperatures on health. A study from Belgium by Demoury et al. analyzed 307,859 natural deaths between 2010 and 2015 (44). They found out that, compared to the normal temperature of 23.1°C, being exposed to cold (-1.7°C 1st percentile and 2.3°C 5th percentile) or high (26.7°C 95th percentile and 31.3°C 99th percentile) temperature significantly increased the mortality risk by 1.32 and 1.21 respectively (44). In this study, despite adjusting to exposure to humidity and fine particulate matter (<2.5 micrometers in diameter, PM_2.5_), the results remained significant. Moreover, women were more vulnerable to heat than men and stronger effects of cold were reported in highly-educated municipalities (44). Not only in Europe, but also in Taiwan/Asia, an analysis of emergency room visits between 2000 and 2014 revealed an association between extreme low temperature and hypertensive events whereas very high temperature increased the risk of visits for kidney disease and ischemic heart disease (45). Higher levels of blood pressure have been associated with cold temperature. In 2003 in Nigeria, Isezuo showed that hypertension-related hospital admissions were significantly more prevalent during the cold season (46). When it comes to chronic kidney disease patients, only one study from China of 109 CKD patients highlighted the seasonal variation of blood pressure and the prevalence of higher blood pressure levels in winter (47). A review of the studies that reported deleterious impact of cold and heat temperatures on population’s cardiovascular health suggested that the damage induced by extreme temperatures might be associated with the activation of the sympathetic nervous system and renin-angiotensin system (48).

Concerning acute kidney injury, there is a need to study seasonal variation of this disease in both developed and developing countries (49). This seasonal variation of acute kidney injury was highlighted in one study from Taiwan (50), two large studies from South Korea (51, 52) and a small Japanese study of 102 patients that analyzed the variation of eGFR in hypertensive patients with CKD (53).

A study from Rome, Italy that analyzed AKI cases between 2010 and 2014 showed a well-defined seasonal pattern with a significant increase of AKI incidence in winter (54) but they demonstrated a significant association with higher humidity levels rather than cold temperature.

On top of heat, cold and humidity, a high atmospheric PM2.5 level could increase lung edema in CKD stage 5 non-dialysis based on a study including 317 patients from Taiwan (55).

Extreme temperature is also important to address in the vulnerable population of children (56) who are mostly susceptible to dehydration during heat and can also have higher morbidity during extreme cold weather.

The study of Obermeyer et al. from USA including 4.8 million individuals showed variation of urea, creatinine and urine specific gravity according to different daily temperatures (57). A recent small study from Ghana showed the seasonal variation of serum creatinine and urea with a significant increase during the hot season (58). Surprisingly, a study from Japan of 903 individuals showed higher levels of urine specific gravity in spring than in summer (59).

In 2016, a public health initiative “ The Peer Kidney Care Initiative” drew attention on the seasonality of cardiovascular outcomes among chronic kidney disease patients and called to address these differences (60). In a Mediterranean climate country, Croatia, authors assessed 135 anuric hemodialysis patients and showed a seasonal variation in most of laboratory parameters like urea, albumin, phosphate, cholesterol and glucose (61).

### Lupus nephritis and seasonal variation

Among nephropathies related to systemic diseases, lupus nephritis was the most related to seasonality (62–65). A study of 41 systemic lupus erythematosus (SLE) patients from Southern France found strong positive correlations between extreme low or high temperatures and renal lupus flares (62). This French group showed significant increase in flares during the spring season whereas patients treated with antimalarials had more flares in the sunny season (62). A review from China highlighted the importance of geographic distribution of SLE and the effect of ultraviolet radiation, climate and altitude on the activity of the disease (63). They reviewed the role of inflammatory mediators, apoptosis and epigenetic factors that are triggered by ultraviolet radiation or cold or humidity and would lead to the inflammatory lesions of systemic lupus erythematosus and in some cases aggravation of lupus nephritis (63). A study of 129 patients in China found a U-shaped relation between the incidence of lupus nephritis flares and environmental temperatures with significant higher cases of membranous nephropathy in December and January (64). A study from the USA of kidney biopsies of 179 SLE patients between 1992 and 2002 showed a significantly higher prevalence of membranous lupus nephritis in winter and spring (65).

### Cold and the kidney

Renal exposure to cold was first experimented during the 60s and 70s in studies from Sweden (66, 67). Eight healthy volunteers exposed to cold showed a decrease in circulating plasma volume, an increase of cardiac output and an increase in the capillary hydrostatic pressure in the renal vessels leading to a reduction in tubular sodium reabsorption and a rise in natriuresis hence hypovolemia and high blood viscosity (67). A study from Israel including 21 healthy volunteers marching at an altitude of 1700 m and temperature of 0°C showed that regular water intake would prevent the dehydration in these conditions (68).

The cold-induced vasoconstriction is due to the activation of the peripheral alpha(2C)-adrenergic receptors (69). Despite the multitude of reports of AKI being more incident in the hot climate, two studies from UK and Japan demonstrate the opposite. The UK study showed a higher trend of AKI and AKI-related mortality during the winter compared to other seasons of the year (70). This trend was observed in community-acquired and in-hospital acute kidney injury incidents (70). Another study including 81,279 AKI patients from Japan showed a higher AKI and severity of AKI cases in January (71).

**Figure 3:**
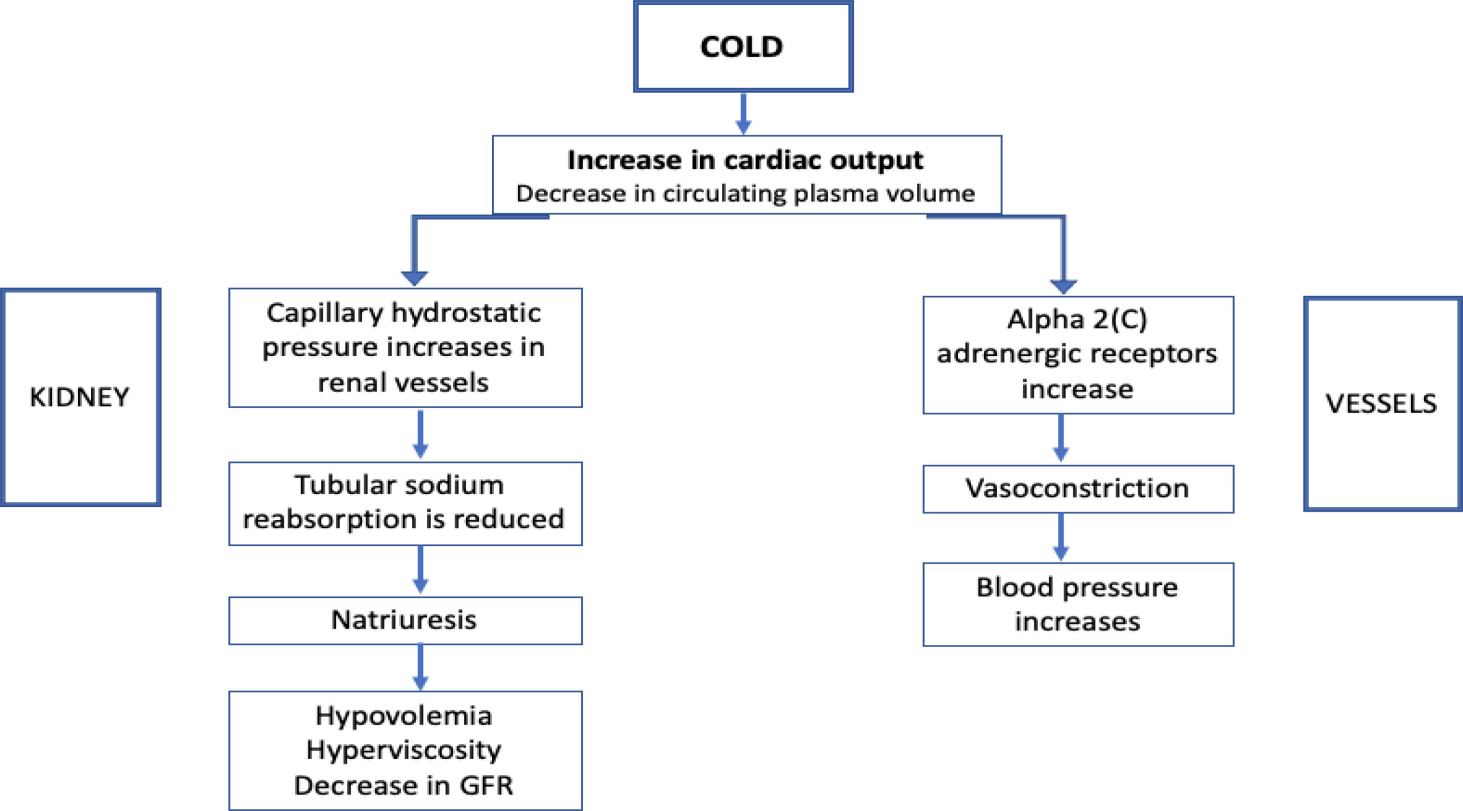
The impact of cold on kidney function and blood pressure.

A Japanese study highlighted the more significant impact of cold on increasing noradrenaline, heart rate and eGFR in normotensive individuals compared to patients with mildly elevated blood pressure. And they suggested to follow the out-of-office blood pressure of these patients to further understand the seasonal change (72). On the opposite, a recent study from Poland evaluated the central blood pressure of 56 patients exposed to cold (10 minutes of -10°C) and found out that the central aortic pressure was higher in hypertensive patients with CKD than in normotensive patients (73). When it comes to hemodialysis patients and seasonal variation, two cohorts from Taiwan showed that hemodialysis patients may have an increase in fluid accumulation during the cold season if they had a lower fractional weight loss (74, 75). Finally, few studies addressed the effect of indoor cold temperatures. Independently of outdoor temperatures, indoor cold temperature can increase nocturia in elderly patients as shown by Saeki et al. from Japan (76). **Figure 3** illustrates the impact of cold on kidney function and blood pressure.

### Heat and the kidney

Fifteen years ago, when the terms “heat” and “kidney” were combined, heatstroke and renal failure from rhabdomyolysis were the first diagnoses to come to mind, more frequently described in men who were exposed to intense exertional activity with few case reports among women (77, 78). But with climate change and global warming, attention has been drawn to the effect of heat waves on chronic kidney injury. A large study from Australia in 2008 showed an increase of kidney disease during the “exposure to excessive natural heat” (79). In 2012, the Thai Cohort Study of 17402 men and 20414 women revealed an association between self-reported kidney disease and self-reported occupational heat exposure (80). In another case-control study from Taiwan, patients followed after 13 years of heat injury revealed an increased risk of CKD in these patients compared to controls (81).

In the first studies from Nicaragua and El Salvador in 2002 about the emerging kidney disease in sugarcane workers, heat exposure was not highlighted. It was a few years later that heat stress and the pathophysiology of chronic recurrent dehydration and hyperuricemia started to be evaluated (82, 83). A total of 189 sugarcane cutters from El Salvador were evaluated for dehydration markers during their strenuous work under very hot temperatures between 39 and 42°C at noon (82).

It is now well believed that heat-related kidney disease could be multifactorial with a higher incidence among disadvantaged population like vulnerable elderly and children, or socio-economically disadvantaged communities (84, 85). In a large Australian study, risk factors for acute kidney injury diagnosed in the emergency room following heat exposure were CKD, heart failure, age>64, male gender, diabetes and hypertension (86). Social and environmental factors seem to be both enhancing the kidney disease of undetermined origin that first emerged from central America and became a topic of research in other parts of the world. Schlader et al. and Chapman et al. have recently reviewed the kidney pathophysiology during heat stress (85, 87). Mostly based on data from animal models, Schlader et al. described the multifactorial mechanism of kidney injury when exposed to heat; heat could induce ischemia and hypoxia at the renal tubular level with ATP depletion which leads to oxidative stress and inflammation and a high risk for acute kidney injury (87). Chapman et al. defined heat stress as the net heat load to which an individual is exposed and results from the interaction of three factors, the environmental heat, the degree of the person’s physical activity that leads to metabolic heat production and the compensatory ability of the body to respond to heat by sweating (85). The combination of exercise and hot temperature can lead to proteinuria that is less likely present during passive heat stress (85). Chapman et al. emphasized two categories of populations exposed to heat stress, those with occupational heat stress like the Mesoamerican nephropathy in young male workers and those exposed to non-occupational heat like the elderly exposed to short days of unusual hot weather (85). The first group will be discussed further in the occupational section and was reported from several countries in the world not only Central American countries like Costa Rica, Guatemala (88–91),, Sri Lanka (92) but also from other developing countries like Thailand, Lebanon, Indonesia and Saudi Arabia (93–96) and even from developed countries mainly the USA (97–100).

Regarding the elderly, the topic is becoming a major public health issue because of the aging of populations globally, the global warming and increase in incidence of heat waves and the susceptibility of elderly kidneys to dehydration (85, 101). Even in countries with northern climate like Canada, a higher risk of acute kidney injury related to heat periods was found in a case-control study including >220,000 older adults of a mean age of 80 (101). In South Korea, an analysis of 21,656 cases of acute kidney injury admitted at the emergency department between 2010 and 2014 showed an increase in risk of AKI with every 1°C of increase in temperature (102).

Some studies could not demonstrate a higher incidence of AKI when heat stress was combined with non-steroidal inflammatory drugs and this association remains controversial (103, 104). A randomized controlled trial that included 40 cyclists with a mean age of 52 years in the US showed a significant increase in serum creatinine and decrease in fractional excretion of urinary sodium before and after one hour of endurance cycling in the heat, but ibuprofen intake did not worsen AKI (103). Another randomized cross-over trial from the US compared ten active males running in the heat versus temperate conditions, both conditions elevated their serum creatinine kinase but urinary neutrophil gelatinase-associated lipocalin (NGAL) was only increased in hot conditions which led to the conclusion that exercising in the heat increases the risk of mild acute kidney injury (105). Passive heat stress might not affect the glomerular filtration rate but the trials are controversial on whether heat acclimation improves or not GFR; a trial from the US showed that permissive dehydration with heat acclimation does not induce an increase in urinary NGAL, another trial from the UK showed that heat acclimatization can reduce AKI incidence but this was not consistent in another study from the US (106–109). Another interesting risk factor for acute kidney injury during heat stress is the consumption of soft drinks during and following exercise (110). This was demonstrated by Chapman et al. in a clinical trial of 12 healthy individuals that were exposed to heat, dehydration and who elevated their uric acid, serum copeptin and urinary NGAL levels after drinking a soft drink compared to water (110).

Few studies have assessed the risk of extreme heat events on patients with established chronic kidney disease. In a large study from the USA that included 7445 end-stage kidney disease patients with a mean age of 61 years, extreme heat was associated with higher mortality and morbidity and this association varied between geographic regions (111). A review of 58,330 hospital admissions during the warm season in Vietnam showed a higher incidence of hospitalization of kidney disease patients and urolithiasis (112). The same was demonstrated in California after assessing all hospitalizations between 1999 and 2009 (113).

**Figure 4:**
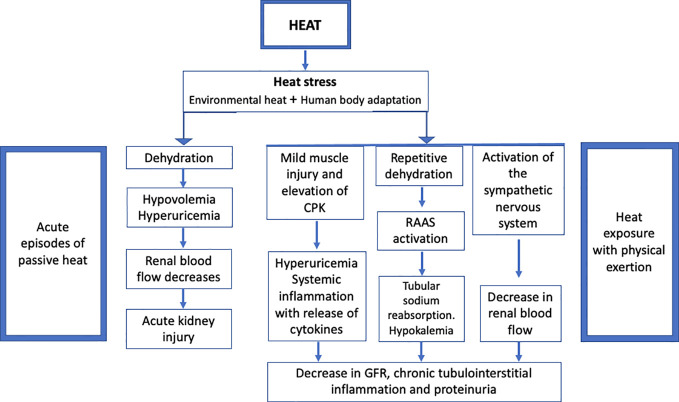
Different consequences of heat on kidney function.

Consequently, apart from the recurrent acute kidney injury secondary to heat stress, it is important to shed light on the risk of kidney stone in hot temperatures because it is expected to substantially increase in the future with the global warming (112, 114). **Figure 4** depicts the different consequences of heat on kidney function.

## Occupation and the kidney

### Occupational safety and health

The Occupational Safety and Health (OSH) has been a crucial component of the International Labour Organization (ILO) since its foundation in 1919. At the beginning, lead toxicity was one of the most important occupational risks that has been addressed. In 1950, after World War II and the awareness about emerging toxic substances, a joint commission ILO/World Health Organization (WHO) convened and defined occupational health (115).

Occupational health should aim at “the promotion and maintenance of the highest degree of physical, mental, and social well-being of workers in all occupations; the prevention among workers of departures from health caused by their working conditions; the protection of workers in their employment from risks resulting from factors adverse to health; the placing and maintenance of the worker in an occupational environment adapted to his physiological and psychological equipment and, to summarize: the adaptation of work to man and of each man to his job” (115).

Occupational health is a discipline that involves many stakeholders, it is adopted differently among countries worldwide and the regulations of OSH are not well followed and implemented in the majority of developing countries. When it comes to occupational-related kidney injury, there are no kidney health-specific recommendations but there are more general recommendations targeting the risk of exposure to heavy metals, infectious agents, chemicals and the environment (116). In addition, there is scarce data at the global level about mortality attributed to occupational-related kidney disease.

### Occupation, socioeconomic status and kidney disease

**Figure 5:**
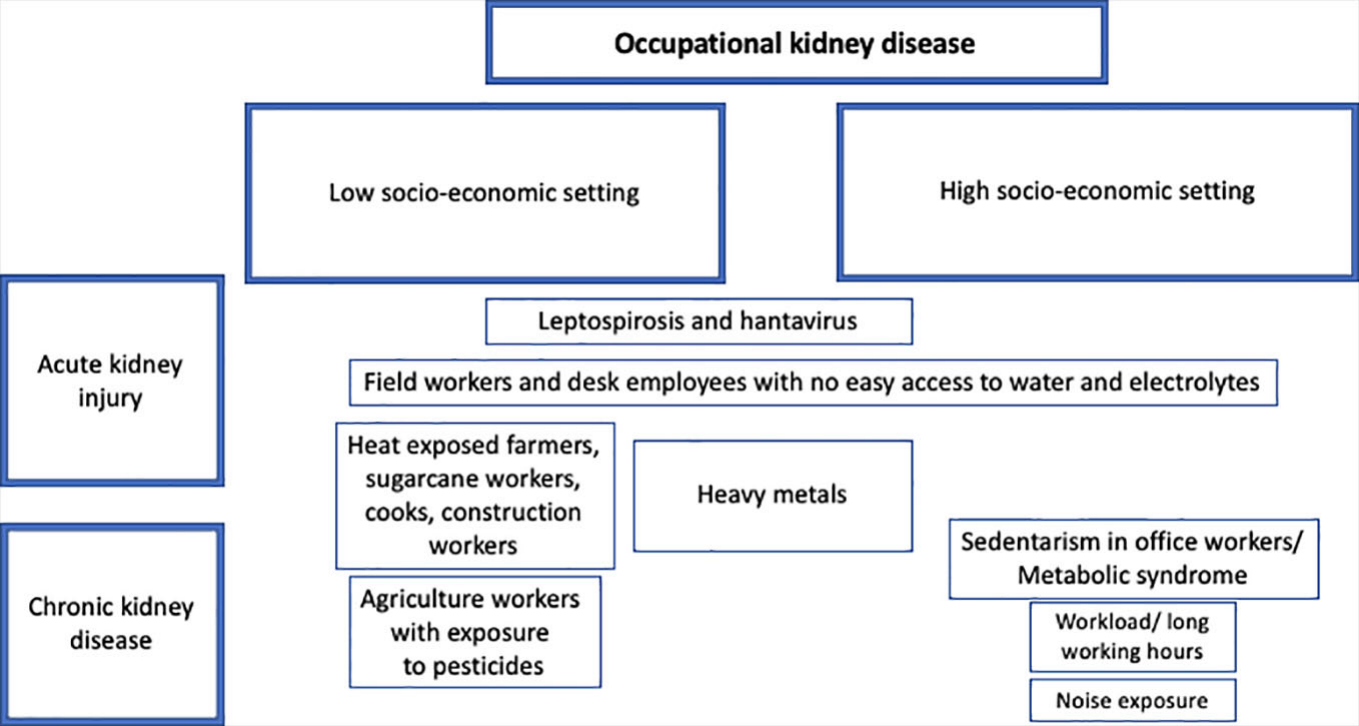
Distribution of occupational factors causing kidney disease across different socio-economic settings.

In most studies evaluating socioeconomic disparities among kidney disease patients, the socioeconomic status (SES) was considered as a combined measure of three indicators, education, income and occupation. A meta-analysis of 43 articles by Zeng et al. showed significant association between CKD prevalence and lower combined SES (117). Lower levels of occupation were also demonstrated to be associated with end-stage renal disease (117). Another meta-analysis by Tao et al. analyzed 14 studies including hemodialysis patients (118). Lower socioeconomic status (SES) indicators were significantly associated with higher mortality in dialysis (118). Moreover, a review of occupational nephropathies highlighted how disadvantaged populations and workers are more exposed to nephrotoxic agents and how poverty is contributing to occupational kidney disease like CKDu in agriculture workers in Central America (119). Finally, a study of 2011 on UK population showed an association between low SES and decreased eGFR. However, the significance of this association was attenuated when SES was adjusted to the components of the metabolic syndrome (120). **Figure 5** illustrates the distribution of different social and environmental occupational factors that cause acute and chronic kidney disease.

### From heavy metals to heat stress: Two eras of occupational kidney disease

Before 2002, the occupational and environmental risk factors that have been linked to kidney disease were mainly heavy metals such as lead, mercury, arsenic, cadmium, uranium and organ solvents such as silica, infectious agents mainly leptospirosis and hantavirus and finally pesticides (121, 122). After the first report about CKD of unknown etiology (CKDu) in agriculture workers in El Salvador in 2002 (123), more than a hundred papers were published about the Mesoamerican nephropathy and heat exposure as a major risk factor. At the same time, several reports from Sri Lanka started to shed light on CKDu in agriculture workers where pesticides seem to be the main risk factor.

### Heavy metals, organ solvents and infectious agents: Old occupational risk factors

Lead and cadmium nephrotoxicity were first described in the 19th century; Lead, cadmium, arsenic and mercury are listed by the World Health Organization as four of 10 chemicals of major public health concern (124). They both cause tubular injury, Fanconi syndrome and may lead to chronic kidney disease (121, 122). Lead causes microcytic anemia, neurologic impairment, gout and kidney disease (122). Most of the first cases of cadmium were described in industrial countries like Belgium and Japan (122). The disease combining anemia, osteomalacia and kidney injury following the ingestion of contaminated rice by cadmium of the mines was called Itai-Itai disease (122). The concurrent toxicity of the two metals lead and cadmium has also been studied with growing evidence about their association with kidney disease, cancer and hypertension (125). Arsenic is found in pesticides and some studies linked kidney injury to urinary arsenic levels, mercury causes tubular and glomerular damage (122). Gold and mercury may also cause auto-immunity (126).

During the last 50 years, the international efforts and the enforcement of strict regulations in developed countries succeeded in minimizing the exposure of communities to lead, cadmium and other heavy metals (127). However, recent studies still report nephrotoxicity of heavy metals despite low levels. A recent analysis of Cadmium accumulation showed a correlation with albuminuria level (128). In 2021, a meta-analysis of 43 studies that analyzed the association of blood lead levels with renal function showed that high BUN is a valuable prognostic test for lead-associated kidney disease (129). A study on selenium from Taiwan and another on mercury from Colombia link the renal toxicity of these metals to genetic predisposition (130, 131).

The long-term toxicity of low levels of heavy metals is still controversial, especially in recent large longitudinal cohorts from high-income countries. Evans et al. studied 10,303 lead-exposed workers followed for 20 years and found no significant association between lead exposure and ESRD (132). Similarly, Steenland et al. found no significant association between higher lead exposure and ESRD in 58307 workers exposed to lead in the USA (133).

Golden et al. reviewed the outcome of 12,400 workers of uranium facilities in the USA between 1930 until 2017 and they found no increased renal mortality (134).

In addition to heavy metals, organic or solvent agents like silica or beryllium have also been evaluated as occupational hazards that may increase the risk of chronic kidney disease (135). High albuminuria was more observed in exposed workers to different solvents such as toluene, hydrocarbons, tetrachloroethene than in non-exposed controls (136). Silica nephropathy is a tubulointerstitial disease mainly, sometimes associated with immune-mediated systemic disease and could lead to end-stage renal disease (137). Occupations exposed to silica are miners, sandblasters, glass manufacturers, masons, ceramic and quarry workers (137).

Concerning infectious diseases, farmers from all over the globe suffered of hantavirus and leptospirosis. In a systematic analysis of 42 studies about the prevalence of Hantaviruses in farmers and forestry workers, the seroprevalence was estimated at 3.7% and 3.8% in these two occupational groups which is significantly higher than the general population (138). Cases of occupational leptospirosis and acute renal failure have been described in low-income countries, in Thailand, India, Bulgaria, Cuba and Argentine (139–143) but also more frequently in high-income countries, in farmers, abattoir workers in Barbados, Taiwan, Japan, Netherlands, Denmark, New Zealand, USA, in a sewage drain worker in Germany and among the French army in France (144–158). Studies on hantavirus and leptospirosis in the Mesoamerican countries were not conclusive even negative (159, 160).

### Occupation, pesticides and CKDu in agriculture workers in Sri Lanka: Old and new story

Since the 1990s, chronic kidney disease of unknown etiology was described in agriculture workers in Sri Lanka. **Table 2** summarizes most of the studies that reported occupational kidney disease in Sri Lanka. The first reports revealed several risk factors for CKDu in these male farmers such as age, drinking contaminated well water, being exposed to pesticides and snake bites (161, 162, 168). More recent studies highlighted as well the role of arsenic and the association between urinary herbicide level and biomarkers of kidney injury (165, 166). In 2016, a series of kidney biopsies in 59 patients with CKDu in Sri Lanka revealed chronic tubulointerstitial lesions with glomerular scarring (163). In 2017, de Silva et al. performed mixed methods research and revealed an association of CKDu with the poorest of the poor marginalized social category in agricultural settlements (164). In 2021, Kulasooriya compared 475 villagers including agriculture workers from endemic and non-endemic regions and demonstrated a significant higher level of heat stress index and urinary neutrophil gelatinase-associated lipocalin (NGAL) in workers from endemic CKDu regions (92). In 2022, Ekanayake et al. studied 188 paddy farmers, factory workers, plantation workers, they showed that Kidney Injury Molecule (KIM-1) is the best urinary biomarker to characterize renal injury in these occupational groups that suffer from CKDu (167).

**Table 2:** Occupational kidney disease in Sri Lanka.

Sri Lanka					
Reference	Type of study	Number of participants	Occupation	Country	Outcome
Wanigasuriya KP, 2007 (161)	Case-control	183 patients with CKDu and 200 controls	Farmers	Sri Lanka	Risk factors: farmer, pesticides, snake bite, drinking well water
Jayasumana C, 2015 (162)	Case-control	125 CKDu patients and 180 controls	Paddy farmers	Sri Lanka, dry zone	Strong association between CKDu and drinking from a well that was abandoned and association between CKDu and spraying glyphosate and other pesticides in paddy fields
Badurdeen Z, 2016 (163)	Cross-sectional	59 patients with elevated creatinine	Farmers	Sri Lanka	Kidney biopsy: acute and chronic interstitial nephritis with glomerular scarring
de Silva MW, 2017 (164)	Mixed methods approach	Historical analyses, a case-control study, ecologic analysis of features of communities and CKDu prevalence, and direct observations and interviews with people in affected communities	Male wage laborer	Sri Lanka	Association of CKDu with the poorest of the poor marginalized social category in agricultural settlements in Medawachchiya
Gobalarajah K, 2020 (165)	Cross-sectional	35 patients	Agriculture workers	Sri Lanka, Northern Province	CKDu showed male preponderance and correlated with arsenic and TDS levels in the drinking water (with spotty pigmentation on hands)
Kulasooriya PN, 2021 (92)	Cross-sectional	475 villagers, 293 agriculture workers in the endemic region and 76 agriculture workers in non-endemic region	Agriculture workers	Sri Lanka	Urinary NGAL biomarker was higher in agriculture workers in endemic CKDu region, the heat stress index was significantly higher in these workers compared to non-agriculture workers and to non-endemic region
Abdul KSM, 2021 (166)	Cross-sectional	210 male and female farmers	Exposure to herbicides in sugarcane farmers	Sri Lanka, Southern province	Higher urinary herbicide levels among sugarcane farmers, potentially linked to the decline in kidney function (ACR, eGFR, and NGAL)
Ekanayake EMDV, 2022 (167)	Cross-sectional	188 participants	Fisherfolk, paddy farmers, sugarcane farmers, factory workers and plantation workers	Sri Lanka	The urinary biomarker KIM-1 is the best to characterize renal injury in occupational groups with CKDu

In 2016, a systematic review of all studies on CKDu conducted in Sri Lanka could not identify a definitive cause and concluded that it was multifactorial, with variable geographic distribution, related to agriculture practices, use of agrochemicals, possible water contamination with heavy metals (169). Another review in 2020 revealed that the CKDu accounted for 70% of CKD cases in endemic areas and it was associated with being a farmer, exposed to heavy metals but found limited data on heat stress (170).

Several authors attempted a comparison of the “chronic interstitial nephritis in agriculture communities (CINAC)” that occurred in Sri Lanka, Central America and other tropical countries (171–173). A systematic review of 26 CKDu studies published in 2016 showed that most of the studies were conducted in Sri Lanka and Mesoamerican countries (171). Other countries reporting CKDu cases were Mexico, India, Tanzania, Tunisia, Japan, USA, Sweden, and Australia. The common risk factors were male sex, agriculture occupation and exposure to heavy metals. In Sri Lanka, agrochemical use was the additional risk factor whereas it was altitude and temperature in the Mesoamerican (171). A deep review by Jayasumana et al. of the CINAC endemic also described the common characteristics of CINAC, such as working in a poor agriculture community, being exposed to agrochemicals, having tubulointerstitial disease with low or absent proteinuria (172). Jayasumana et al. shed light as well at the different main triggers reported so far in the literature, the pesticide and heavy metal exposure from one side and the heat stress on the other (172). They argued that the absence of CINAC in some other hot areas of Sri Lanka, Cuba and Myanmar and the presence of this disease in women and children could not support heat stress and dehydration as a main trigger but it may be an additive on occupational and environmental toxins (172).

### Heat stress as occupational risk for kidney disease

#### Heat and kidney stones

Before the era of CKDu and Mesoamerican nephropathy, heat exposure during work was considered as a risk factor for kidney stones. Warm climates at work, insufficient fluid intake and improper access to restrooms and bathrooms could lead to nephrolithiasis. In a recent review on occupational kidney stones, Malieckal et al. highlighted the several causes such as dehydration while working outdoors in hot temperatures, the risk of metabolic syndrome and uric acid stones in sedentary jobs and risk of calcium mobilization from bone in astronauts when working without gravity (174).

### The Mesoamerican nephropathy

A growing attention in the last two decades took place towards the physical exertion of sugarcane cutters in very hot temperatures during the harvest season in El Salvador, Nicaragua, Guatemala and Costa Rica. Although the first reviews from El Salvador emphasized the role of pesticides (123), papers that were later published analyzed all possible causative factors of CKD in agriculture workers and specifically sugarcane workers in the four countries of Central America (82, 88, 90, 175–218). **Table 3** summarizes these findings. The first research workshop on Mesoamerican nephropathy took place in 2012 in Costa Rica and paved the path of coordination between regional and international initiatives in order to further elucidate the cause of the disease and address it with interventional actions (219). In addition to research collaboration, in 2009, facing the high burden of farmers on dialysis in El Salvador (50.7%), El Salvador’s Ministry of Health collaborated with Cuba’s Ministry of Public Health and the Pan American Health Organization (PAHO) to launch a cooperative initiative to face the endemic kidney disease (220). Among 29 papers from Nicaragua, 13 from el Salvador, 4 from Costa Rica and 4 from Guatemala, sugarcane workers in the hottest areas suffered from kidney injury. Hansson et al. showed that, in comparison to banana, rice, coffee cultivation, the sugarcane cultivation had the highest burden of CKD (218). Kidney biopsies from patients in Nicaragua and El Salvador confirmed the chronic tubulointerstitial lesions and glomerular ischemia (185, 205, 207). Raines et al. found low eGFR in half of 151 male agriculture workers in Nicaragua, proteinuria of 300mg in <10%. Risk factors were pesticide exposure, long working hours and sugarcane chewing (179). Several studies demonstrated also a significant association between heat, dehydration during the harvest season and biomarkers of kidney injury in Nicaragua (178, 180, 183–185, 189, 192, 197, 199), El Salvador (82, 211), Guatemala (213) and Cost Rica (88, 215–218).

**Table 3:** Distribution of occupational kidney injuries among Mesoamerican countries.

Low and middle-income countries					
Central America					
Reference	Type of study	Number of participants	Occupation	Country	Outcome
Torres, 2010 (175)	Cross-sectional	479 men and 617 women	Sugarcane workers and farmers	Nicaragua	High prevalence of CKD in male workers (18%)
Sanoff SL, 2010 (176)	Case-control	124 cases of renal insufficiency and 873 controls	Agriculture workers	Nicaragua	Agriculture and drinking more than 5 l of water per day was associated with renal insufficiency
Laux TS, 2012 (177)	Cross-sectional	293 participants	Coffee-farmer	Nicaragua	High CKD prevalence including high prevalence of microalbuminuria
Ramirez-Rubio O, 2013 (178)	Qualitative study	19 interviews	Male manual laborers	Nicaragua	Diuretics, antibiotics and NSAIDs in combination with dehydration can be the cause of AKI
Raines N, 2014 (179)	Cross-sectional with nested case-control analysis	424 individuals (151 worked in agriculture)	Agriculture, sugarcane workers	Nicaragua	Low eGFR was present in 41.9% of men. Proteinuria of 300 mg in <10%. NSAIDs not associated with decreased eGFR. Risk factors found: duration of cutting sugarcane during dry season, pesticide exposure, sugarcane chewing
Laws RL, 2015 (180)	Observational	284 workers	Sugarcane workers	Nicaragua	Decrease in eGFR over 6-month period moslty in seed cutters (loss of 8.6 mL/min)
Ramírez-Rubio O, 2016 (181)	Cross-sectional	200 adolescents	Prior to work in a high endemic region	Nicaragua	High levels of kidney injury biomarkers
Laws RL, 2016 (182)	Longitudinal cohort	284 workers	Sugarcane workers	Nicaragua	High urinary NGALs during the harvest associated with high decrease in eGFR over 6 months
Wesseling C, 2016 (183)	Cross-sectional	29 males	Sugarcane workers	Nicaragua	Remarkable decrease in eGFR after 9 weeks of harvest
Wesseling C, 2017 (184)	Cross-sectional	194 male workers	Sugarcane workers compared to construction workers and small-scale farmers	Nicaragua	Heat stress, dehydration and kidney dysfunction associated with sugarcane cutters
Riefkohl A, 2017 (159)	Cross-sectional	282 workers: 47 sugarcane and 160 in other industries	Sugarcane workers	Nicaragua	Analysis of leptospirosis association with CKDu in workers was not conclusive
Wijkström J, 2017 (185)	Case series	19 cases with kidney biopsies	Men working in sugarcane cultivations	Nicaragua	Kidney biopsies: glomerular ischemia and glomerulosclerosis, tubular atrophy, interstitial fibrosis and mild vascular changes
Kupferman J, 2018 (186)	Cross-sectional	326 participants	Sugarcane workers	Nicaragua	High incidence of AKI and half develop CKD
Gonzalez-Quiroz M, 2018 (187)	Longitudinal, 2-year follow-up	263 men and 87 women	Agriculture	Nicaragua	Agriculture work and lack of shade at work associated with rapid kidney function decline in men
Yih WK, 2019 (160)	Case-control	320 participants tested for leptospirosis or hantavirus	Miners or construction workers	Nicaragua	High CKDu in miners and construction workers. No evidence of a causal link between infectious cause and CKDu
Gallo-Ruiz L, 2019 (188)	Prospective	224 male and female workers	Brick workers	Nicaragua	High prevalence of CKD in brick workers with 100% of cases being male
Hansson E, 2019 (189)	Cross-sectional	545 workers	Sugarcane workers	Nicaragua	Highest workload associated with more kidney damage
Petropoulos ZE, 2020 (190)	Observational	251 male workers	Sugarcane Workers	Nicaragua	Workers reporting urinary-related symptoms had higher kidney injury biomarkers and positive leucocyte esterase on dipstick without urinary infection
Ferguson R, 2020 (191)	Cross-sectional	1227 participants	Sugarcane workers	Nicaragua	CKDu associated with current or past work in sugarcane industry
Fischer RSB, 2020 (192)	Case-control	18 renal case patients and 36 controls	Agriculture workers exposed to trace elements	Nicaragua	Nickels were higher in toenails from cases
Smpokou ET, 2020 (193)	Nested case-control	350 young adults followed over 2 years	Sugarcane Workers	Nicaragua	No difference in pesticide levels between those who had a decline in kidney function and those who had stable kidney function
Glaser J, 2020 (194)	Interventional study	Harvest 1: 427 workers Harvest 2: 488 workers	Sugarcane workers	Nicaragua	Interventions better implemented to burned cane cutters than seed cutters with improvement in GFR loss and less incident acute injury after interventions in burned cane cutters
Leibler JH, 2021 (195)	Cross-sectional	210 children	Prior to occupational exposure	Nicaragua	Subclinical kidney injury
Petropoulos ZE, 2021 (196)	Cross-sectional	Temperatures during harvest months between 1973 and 2014	Sugarcane workers in heat	Nicaragua	The sugarcane company with endemic CKDu is located in one of the consistently hottest regions of the country.
Pacheco-Zenteno F, 2021 (198)	Qualitative study	21 key informants of low and middle management involved in implementing interventions to reduce heat stress	Sugarcane workers	Nicaragua	Prioritizing production over health seemed to be related to the implementation challenges
Stallings TL, 2021 (197)	Cross-sectional	242 workers	Sugarcane workers with heat	Nicaragua	Working as a cane-cutter associated with dysuria
Hansson E, 2022 (199)	Cross-sectional	30 cases of AKI and 53 workers with stable creatinine	Sugar cane	Nicaragua	Serum creatinine increase correlated with urinary biomarkers of kidney injury (like KIM1)
Keogh SA, 2022 (200)	Cross-sectional	569 cases	Agriculture, sugarcane, corn, plantain, brickmaking, and road construction industries	Nicaragua, El Salvador	CKD prevalence highest in Salvadoran sugarcane (14.1%), then Salvadoran corn (11.6%), and Nicaraguan brickmaking (8.1%). Nicaraguan sugarcane had the lowest prevalence, likely due to screenings prior to employment
Andersson A, 2022 (201)	Cross-sectional	458 workers	Sugarcane cutters tested before and at the end of harvest seasons	Nicaragua, El Salvador	Increases in creatinine reflects reduction in GFR as estimated by eGFR based on Cystatin C
Trabanino RG, 2002 (123)	Observational	205 cases of ESRD	Farmers	El Salvador	Suspicion of pesticides as a cause of ESKD in farmers
Gracia-Trabanino R, 2005 (202)	Observational	291 men	Farmers	El Salvador	High prevalence of CKD in male farmers; low-grade proteinuria; pesticides not associated
Orantes CM, 2011 (203)	Cross-sectional	343 men, 432 women	Farmers	El Salvador	High prevalence of CKDu and male farmers have occupational and traditional risk factors
Peraza S, 2012 (204)	Cross-sectional	256 men, 408 women	Sea-level current sugarcane and past cotton production vs 500 m altitude-sugarcane, coffee, and service-oriented workers	El Salvador	Higher prevalence of CKD (30%) in the sea-level workers vs 4% in those at 500 m
Wijkström J, 2013 (205)	Case series	8 cases with kidney biopsies	Men working in plantations	El Salvador	CKD, low-grade albuminuria and glomerular ischemia, tubular atrophy and interstitial fibrosis
Vela XF, 2014 (206)	Cross-sectional	223 men and women	Farmworkers exposed to agrochemicals	El Salvador	Prevalence of CKD 50%
López-Marín L, 2014 (207)	Cross-sectional	46 kidney biopsies of CKDu	Agriculture workers	El Salvador	Histology: interstitial fibrosis and tubular atrophy
Orantes CM, 2014 (208)	Cross-sectional	976 men and 1412 women	Agriculture workers exposed to agrochemicals	El Salvador	CKD associated with male sex, agricultural occupation, and contact with the agrochemical methyl parathion.
García-Trabanino R, 2015 (82)	Cross-sectional	189 workers	Sugarcane cutters under heat stress	El Salvador	Recurrent dehydration associated with reduced eGFR
Herrera Valdés R, 2015 (209)	Descriptive study	10 women with CKDu	Agriculture/Exposed to agrochemicals	El Salvador	Tubulointerstitial nephropathy
Orantes Navarro CM, 2015 (210)	Cross-sectional	1412 women from disadvantaged populations	Exposed to agrochemicals	El Salvador	Prevalence of CKDu of 13.9%; multifactorial CKD (traditional and occupational causes)
Wegman DH, 2018 (211)	Interventional study, implementing water, rest and shade (WRS)	80 workers	Sugarcane workers	El Salvador	The group with WRS intervention had eGFR loss of -3.4 mL/min vs -5.3 mL/min in the control group
Laux TS, 2016 (212)	Cross-sectional	242 patients on hemodialysis	Agriculture workers	Guatemala	Lower prevalence of CKDu in Guatemala compared to El Salvador among dialysis patients
Butler-Dawson J, 2018 (213)	Observational	330 workers	Sugarcane cutters	Guatemala	36% had decline in kidney function across harvest
Sorensen, 2020 (90)	Interventional study, water, electrolytes, rest and shade (WERS)	517 workers	Sugarcane workers	Guatemala	Interventions can stabilize the decline in eGFR
Butler-Dawson, 2021 (214)	Cross-sectional	283 workers	Sugarcane workers	Guatemala	Discrepancy between self-reported smoking (12%) and urinary cotinine levels (34%)
Crowe J, 2013 (215)	Observational	Assessment of occupational heat	Sugarcane harvesters	Costa Rica	High level of heat stress
Crowe J, 2015 (216)	Cross-sectional	106 harvesters vs 63 non-harvesters	Sugarcane workers	Costa Rica	High prevalence of dehydration and heat illness among harvesters
Wesseling C, 2015 (217)	Analysis of CKD mortality	From 1970 to 2012	Sugarcane workers in Guanacaste province	Costa Rica	Increased CKD mortality in males working in sugarcane production in Guanacaste compared to the rest of Costa Rica
Crowe J, 2022 (88)	Observational	27 field workers and 45 non-field workers	Heat exposed field rice workers	Costa Rica, Guanacaste	Lower eGFR among field workers associated with heat stress
Hansson, 2021 (218)	Ecological study	5 municipalities	Sugarcane cultivation in heat compared to banana, rice and coffee cultivation	Five countries: Mexico, Guatemala, El Salvador, Nicaragua, Costa Rica	High CKD burden in hot areas with sugarcane cultivation

Jayasumana et al. argued that heat stress is not the major trigger of kidney disease in sugarcane cutters (174). Ordunez et al. agreed on that point because they found high CKD mortality trends between 1997 and 2003 in Central American countries among women and children in addition to men (221). In addition, González-Quiroz M et al. analyzed 25 epidemiological studies and found no association with heat stress or pesticide exposure with CKDu in Meso-America (222). However, Wesseling et al. exposed all the evidence that primarily links heat stress to this endemic kidney disease (223). They explained that strong-design studies demonstrated a decline in kidney function across the harvest due to heat and workload and that interventions implementing water, rest and shade (WRS) halted the decline in kidney function in these workers in Nicaragua (223). Other studies highlighted the risk of occupational heat strain on kidney health of workers (224, 225). A systematic review of 111 studies performed in 30 countries included 447 million workers with 40 different jobs, defined heat stress as wet-bulb globe temperature above 22°C or 24.8°C and showed that working under heat stress increased by 4 times the risk of heat strain; 15% of these individuals experienced kidney injury or disease (225). A review of farmworkers across the globe showed how heat stress affects all aspects of health and specifically kidney health in agriculture workers (226). Hansson et al. suggested in a detailed review the pathophysiology of heat stress causing kidney disease in agriculture workers (227). They discussed how systemic and kidney inflammation triggers such as endotoxemia, sub-rhabdomyolysis muscle damage, sugar intake, hyperuricemia and decreased renal blood flow could contribute to the acute kidney injury (228).

### Occupational kidney disease in other low and middle-income countries

In addition to Central America and Sri Lanka, hundreds of occupational renal disease studies were reported after 1990 -excluding heavy metals- in other low and middle-income countries (**Table 4**), like India, Thailand, China, Indonesia and Nepal in Asia, Egypt, Kenya, Malawi, Ghana, Nigeria, Morocco and South Africa in the African continent, Lebanon, Turkey and Iran in the Middle-East, Bulgaria in Europe, Mexico in North America and Cuba, Argentine and Brazil in South America (22, 80, 94, 95, 233–261).

**Table 4:** Distribution of occupational kidney injuries among low and middle-income countries.

Low and middle-income countries						
Reference	Type of study	Number of participants	Occupation	Country	Outcome
**South-East Asia**					
Ittyachen AM, 2007 (139)	Retrospective	53 confirmed cases of leptospirosis	Occupational exposure	India	Leptospirosis-associated renal failure
Singh S, 2011 (229)	Cross-sectional	70 workers	Workers exposed to pesticides	India	DNA damage and nephrotoxicity in those exposed to pesticides
Singh A, 2016 (230)	Cross-sectional	94 kitchen workers and controls	Kitchen workers exposed to polycyclic aromatic hydrocarbons (PAHs) emissions in indoor air of commercial kitchen	North India	PAHs were above the norms in all air analyses of kitchens; higher albuminuria levels in kitchen workers
Mohanty NK, 2020 (231)	Cross-sectional	2978 people screened for CKD	Agricultural workers	India	CKD was highly prevalent with 48% working in agriculture and 49% drinking from wells
Farag YMK, 2020 (232)	Cross-sectional	1201 participants	Farmers	India	“Uddanam nephropathy”: working as a farmer was strongly associated with the high prevalence of CKD (32.2%)
Venugopal V, 2020 (228)	Cross-sectional	340 workers	Steelworkers with heat exposure and moderate to heavy labour	India	Years of exposure to heat were significantly associated with risk of kidney stones
Venugopal V, 2021 (233)	Cross-sectional	1500 workers	Heat-exposed workers	India, South	Occupational heat exposure associated with reduction in kidney function
Venugopal V, 2021 (234)	Cross-sectional		Informal workers exposed to heat	India	Research challenges in assessing the association between occupational heat exposure and kidney disease
Nainggolan G, 2021 (95)	Cross-sectional	119 subjects	Shoe factory with heat exposure	Indonesia	Urine density predicts kidney damage
Fitria L, 2020 (22)	Cross-sectional	354 male farmers	Rice farmers	Indonesia	CKDu (24.9%) was associated with the altitude of the farm and with the longer use of insecticides
Tangkanakul W, 2000 (140)	Retrospective	59 cases of Leptospirosis and 118 controls	Applying fertilizer in wet fields, plowing in wet fields and pulling out rice plant sprouts in wet fields	Thailand	Leptospirosis-associated renal failure
Honda R, 2010 (235)	Cross-sectional	795 residents	Rice farmers exposed to cadmium in rice and water	Thailand	Increased urinary beta(2)-microglobulin and N-acetyl-beta-d-glucosaminidase associated with the increase in urinary cadmium
Tawatsupa B, 2012 (80)	Thai Cohort Study	37816 workers	Jobs with heat stress	Thailand	Occupational heat-stress associated with self-reported kidney disease. Physical jobs are at higher risk than office workers (both with heat stress)
Mueangkhiao P, 2020 (236)	Cross-sectional	59 farmers	Farmers exposed to pesticides	Thailand	Occupational exposure to pesticides (glyphosate mostly used) linked with kidney injury
Arphorn S, 2021 (237)	Cross-sectional	65 female rice farmers	Rice farmers	Thailand	Dipstick analysis showed high urine density (38.5%) and proteinuria (21.5%)
Devkota HR, 2020 (238)	Qualitative	25 men	Migrant workers from Gulf countries and Malaysia	Nepal	Migrant workers reported more kidney failure
**Western Pacific**					
Yang HY, 2011 (239)	Case-control	40 cases and 98 matched controls	Manufacturing Chinese herbal medicine	China	Manufacturing and selling Chinese herbal medicine associated with kidney failure
Wang T, 2011 (240)	Cross-sectional		Chromate exposure	China	Blood and urine chromate associated with kidney damage
Ou S, 2017 (241)	Cross-sectional	10 patients	Chemical fiber factory exposed to carbon disulphide	China	High level of proteinuria and mesangial and nodular hyperplasia on biopsies
Shen Q, 2019 (242)	Cross-sectional	1713 adults	All occupations	China	Low socioeconomic status especially of occupational nature related with high CKD prevalence
Zhang S, 2020 (243)	Cohort	6869 steelworkers	Steelworkers	China	Long-term night-shift work associated with early stage of kidney dysfunction
Yang X, 2020 (244)	Case report	One man	Long distance running	China	Acute tubular necrosis
**Africa**					
Mourad BH, 2020 (245)	Cross-sectional	29 male workers	Silica-exposed workers	Egypt	Urinary silicon levels were significantly correlated with urinary KIM1 levels
Mwangi DM, 2009 (246)	Cross-sectional	33 silica-exposed male factory workers	Ceramics, bricks and tiles factory	Kenya	Associated subclinical tubular and glomerular damage in silica-exposed workers
Hamilton SA, 2020 (247)	Cross-sectional	821 adults	57% of the sample: agriculture workers	Malawi	No CKD similar to the CKDu in agriculture workers of Mesoamerica or SriLanka
Adjei, 2019 (248)	Observational	2492 adults	All occupations	Ghana	CKD was not associated with occupational level or any component of the socioeconomic status
Ngajilo D, 2017 (249)	Case report		Exposure to inhalation of thinners	South Africa	Acute tubular necrosis and rhabdomyolysis
Okaka, 2014 (250)	Retrospective	1278 patients	Unskilled workers	Nigeria	43% of unskilled workers among dialysis patients
Ben Khadda, 2022 (251)	Cross-sectional	431 patients	Agriculture	Morocco	CKD strongly associated with agriculture work and contact with agrochemicals
**Europe**					
Christova I, 2003 (141)	Retrospective	455 confirmed cases of leptospirosis	Fishing and livestock farming	Bulgaria	30.3% of occupational origin and 33.8% associated with renal failure
**Middle East**					
Balali-Mood M, 2010 (252)	Cross-sectional	108 men	Traditional tile workers exposed to lead	Iran	No correlation between blood level concentration and GFR
Doueihy C, 2022 (94)	Case-control	238 hemodialysis patients and 238 matched controls	Heat-exposed cooks, construction workers	Lebanon	Outdoor and indoor heat-exposed occupations were associated with end-stage kidney disease
Aksoy N, 2020 (253)	Cross-sectional	393 kidney transplant recipients	All occupations	Turkey	Self-employed and public service officers significantly associated with end-stage kidney disease diagnosed after the age of 32
**North America**					
González-Yebra AL, 2006 (254)	Cross-sectional	50 shoe-workers and 25 controls	Toluene-exposed shoe workers	Mexico	Urinary albumin and N-acetyl-beta-D-glucosaminidase were associated with toluene exposure
Christensen DL, 2014 (255)	Clinical trial	10 male Tarahumara runners	Runners for 78 Km	Mexico	Reversible kidney damage after 48-hour rest
Pérez-Herrera N, 2019 (256)	Cross-sectional	41 working children from Ticul community who work in shoe manufacturing	Footwear manufacturing with benzene exposure	Mexico	Children exposed to benzene had high trnas, trans-muconic acid and proteinuria
Aguilar-Ramirez D, 2021 (257)	Cross-sectional	579 participants	Agriculture	Tierra Blanca, Mexico	Lower eGFR significantly associated with agriculture workers
López-Gálvez N, 2021 (258)	Cross-sectional	101 migrant workers (half worked in an organic field) and 50 controls	Migrant farm workers exposed to heat and pesticides	Mexico	eGFR was significantly lower in the migrant workers in conventional field
Díaz de León-Martinez L, 2021 (259)	Cross-sectional	Children from Ticul community	Occupational exposure to polycyclic aromatic hydrocarbons	Mexico	Early kidney damage (high urinary NGAL)
**South America**					
Roura Carrasco J, 1992 (142)	Retrospective	215 cases of leptospirosis	Young male farmers	Cuba	Acute kidney failure, the main complication
Vanasco NB, 2008 (143)	Retrospective	186 cases of confirmed leptospirosis	Rural occupations	Argentine	Acute renal failure
Nerbass FB, 2019 (260)	Clinical trial	14 males exposed to heat and 17 controls	Indoor foundry workers near furnaces with heavy clothing and 8.5 hour/day work shift	Brazil	Workers exposed to heat stress (>=28.9 degrees C) had a greater decline in estimated glomerular filtration rate compared to controls
Prudente IRG, 2021 (261)	Cross-sectional	208 illiterate male workers	Rural workers exposed to agrochemicals	Brazil	Lower GFRs and lower butyrylcholinesterase levels correlated with the longer duration of exposure to organophosphates (pesticides)

In 2014, Almaguer et al. reviewed countries that reported CKDu in agriculture workers and added India and Egypt to the list of Mesoamerican countries and Sri Lanka. They found that these countries have spots of high prevalence of chronic tubulointerstitial disease in men exposed to pesticides and dehydration (262). A review of CKDu in India found fragmented reports without clear characterization of the disease. It seems different from the Mesoamerican and Sri Lanka nephropathy as it affects older patients and it is associated with low levels of proteinuria and mild hypertension (263). But data evolved differently lately. In 2020, Mohanty et al. screened 2978 individuals in India and found a prevalence of 48% of CKD in agriculture workers and 49% were drinking from wells (231). The same year, Farag et al. found a CKD prevalence of 32.2% among 1201 participants and the “Uddanam nephropathy” was strongly associated with the farmer occupation (232). Venugopal et al. extensively studied as well he link between occupational heat exposure, kidney stones or kidney dysfunction in heat-exposed workers in the South of India (228, 233, 234). In the North of India, there has been a report about kitchen workers and higher albuminuria in those exposed to indoor air hydrocarbons in commercial kitchens (230). Papers from Indonesia reported the risk of kidney damage in shoe factory workers and rice farmers (22, 95). In Thailand, the Thai Cohort Study of 37816 included workers showed an association between occupational heat stress and self-reported kidney disease (80). And in Nepal, there is a serious concern about the high incidence of kidney disease among Nepali workers who migrated to work in the Gulf countries or Malaysia, which is still under research (238). In China, the reports on occupational kidney disease are different, do not include agriculture workers but they highlighted in 1713 adults the strong association between low SES and CKD prevalence (242). Another experimental paper from China studied the molecular alterations caused by the most used herbicide, glyphosate. Glyphosate, by altering metabolic pathways and increasing oxidative stress can cause various diseases like cancers, Parkinson’s disease and renal dysfunction (264).

In the African continent, reports from Malawi and Ghana ruled out the association between CKD, occupation, agriculture and socioeconomic status (247, 248). Morocco showed a high association between agriculture work and CKD (251). Nigeria reported in a review of 1278 patients 43% prevalence of unskilled workers among dialysis patients (250). In Kenya, workers in ceramics, bricks and tiles factory, silica-exposed had subclinical kidney injury (246).

In the Middle-East, a multicenter case-control study of 476 patients in Lebanon showed that heat-exposed cooks and construction workers were at high risk for end-stage kidney disease (94).

In the Americas, Mexico is also another hotspot for CKDu as studies from Tierra Blanca have shown 25% prevalence of CKD and a majority of non-traditional etiology (257, 258, 265). CKDu in Mexico seems to be more prevalent in poor communities (266).

Papers from Brazil emphasized the association between kidney disease and pesticides (261). A systematic review of Prudente et al. showed a high risk of renal injury in workers exposed to organophosphates and herbicides (267). Another interesting paper from Brazil showed how indoor foundry workers under extreme heat have a greater decline in their glomerular filtration rate compared to controls (260).

### Occupational kidney disease in high-income countries

High-income countries that reported cases of occupational renal disease between 1990 and 2022 are USA and Barbados in North America, New Zealand, Australia, Japan, South Korea, Taiwan in the Western Pacific, UK, Ireland, France, Germany, Spain, Italy, Poland, Netherlands, Norway and Denmark (97, 98, 268–300) (**Table 5**).

**Table 5:** Distribution of occupational kidney injuries among high-income countries.

High-income countries											
Reference	Type of study		Number of participants		Occupation		Country		Outcome		
**North America**										
Gale DA, 1990 (144)	Retrospective		177 patients with positive serology for leptospirosis		Agricultural workers (35%), labourers (24%) and non-manual outdoor workers (19%)		Barbados			Leptospirosis-associated acute renal failure	
Steenland NK, 1990 (268)	Case-control study		325 men with ESKD with matched controls		All occupations		Michigan, USA			High risk of ESKD with occupational exposure to solvents (cleaning agents and degreasers) and silica exposure in foundries or brick factories or during sandblasting	
Vupputuri S, 2012 (269)	Case-control study		504 newly diagnosed CKD cases and 457 controls		Occupational silica exposure		Georgia, USA			Significant association between duration of exposure to silica and CKD	
Sponholtz TR, 2016 (270)	Case-control study		547 newly diadnosed CKD patients and 508 controls		Assessing exposure to endotoxins and fine particles		North Carolina, USA			Crop production in agriculture associated with increased CKD. Exposure to dusty conditions associated with increased risk of glomerulonephritis across industry.	
Moyce S, 2016 (271)	Observational study		295 workers		Agriculture workers		California, USA			Incident AKI detected in 11.8% of workers after a single summer day	
Moyce S, 2017 (272)	Observational study		283 participants		Agriculture workers		California, USA			12.3% of incident AKI, increased risk with heat	
Kupferman T, 2017 (145)	Case-report		3 cases		Abattoir workers		USA, New York			Renal failure with leptospirosis	
Mix J, 2018 (273)	Cross-sectional		192 workers		Agricultural workers		Florida, USA			A high prevalence of dehydration (53%) and AKI (33%)	
Murray, 2019 (274)	Cross-sectional		19 participants in focus groups, 75 current taxi drivers, 25 controls		Taxi drivers		USA			Drivers reporting health concerns among these concerns: kidney disease	
Chapman, 2020 (275)	Experimental		13 healthy adults		Exposed to heat stress and dehydration		USA			Risk of AKI higher in humans with large magnitudes of hyperthermia and dehydration during physical work in the heat and limiting the hyperthermia and/or dehydration reduces the risk of AKI	
Moyce S, 2020 (276)	Cross-sectional		445 participants		Agriculture workers on farms		California, USA			The increased volume of water that workers drank was associated with high odds of acute kidney injury	
Moyce S, 2020 (277)	Cross-sectional		357 participants		Agriculture workers in two farms during summer harvest seasons		California, USA			Heavy occupational workload associated with acute effects on renal health	
Boyle SM, 2020 (278)	Cross-sectional		23,692		All occupations categorized by income		Philadelphia, USA			Lower neighborhood socioeconomic index (a composite assessment of neighborhood income, educational attainment, and occupation) was associated with a higher risk of CKD	
Smith, 2022 (97)	Cross-sectional		50 participants receiving frequent emergent-only hemodialysis		Undocumented workers, 66% from Mexico		Georgia, USA			Occupations identified: using pesticides in landscaping, agriculture and heat exposure, construction, dry cleaning, and lead paint fumes in construction	
Shi DS, 2022 (98)	Cross-sectional		608 heat-related AKI cases		Heat exposure indoor and outdoor (industries)		USA			Indoor and outdoor industries: manufacturing, construction, mail and package delivery, and solid waste collection. 95.2% were male, 50.0% with hypertension	
Shrestha D, 2022 (279)	Observational		36703 male autoworkers		Autoworkers		USA			Modest associations between metalworking fluids and ESRD classification of glomerulonephritis and diabetic nephropathy	
**Western Pacific**										
Vickery B, 2006 (146)		Retrospective		15 cases of leptospirosis		Meat and agriculture workers		New Zealand		14 out of 15 had renal failure
Rao N, 2020 (280)		Case report		26-year-old man		Stonemason exposed to silica		Australia		ANCA vasculitis and IgA nephropathy
Yamashita H, 2010 (147)		Case reports		Two patients		Rice farm work		Japan		Leptospirosis-associated acute renal failure
Tsurugano S, 2012 (281)		Cross-sectional		1231 male office workers		Job stress		Japan		Association between job stress, metabolic syndrome, high blood pressure and low eGFR
Noborisaka Y, 2013 (282)		Retrospective		3964 men and 2698 women		Middle-aged workers/employees (all types)		Japan		Development and progression of CKD was related to hypertension, obesity, diabetes and miscellaneous job category: security guards, farmers and housekeeping
Lee J, 2016 (283)		Prospective cohort		1744 dialysis patients		All occupations		South Korea		Mechanician, laborer and farmer were the occupations with significant late referral to a nephrologist. Late referrals had lower hemoglobin, higher phosphate and less controlled blood pressure at the dialysis initiation
Lee, 2020 (284)		Cross-sectional		20,851 workers >=20 years		All occupations		South Korea		Long working hours associated with decreased eGFR
Kim, 2021 (285)		Cross-sectional		17,154 participants		Noise exposure		South Korea		High CKD prevalence associated with females who experienced long-term occupational noise (≥ 240 months)
Yang HY, 2005 (148)		Case-control		22 cases of confirmed leptospirosis and 21 controls of suspected cases		Occupations exposed to leptospirosis		Taiwan		Most had acute renal failure; 10 out of 22 associated with occupational exposure
Chen SC, 2013 (286)		Case-control		441 occupational drivers and 432 controls		Occupational drivers		Taiwan		Metabolic syndrome and albuminuria more prevalent among drivers
Wu CF, 2015 (287)		Cross-sectional		44 workers		Melamine tableware manufacturing factories		Taiwan		Ambient melamine exposure increases the levels of urinary β2-microglobulin and N-acetyl β-d-glucosaminidase (NAG) levels
Chuang KJ, 2015 (288)		Case-control		66 welding workers and 12 controls		Welding workers exposed to metal fume		Taiwan		PM2.5 was significantly higher in welding workers. Urinary NGAL was significantly higher in welding workers
Wang HK, 2020 (149)		Cross-sectional		57 cases of Leptospirosis		Occupational contact with soil		Taiwan		30% of AKI
**Europe**										
Pai P, 1998 (289)		Cross-sectional		47 workers with and 112 workers without protective equipment and a control group of 92 non-exposed		Occupational hydrocarbon exposure in paint sprayers from a car manufacturing plant		UK, Northwest of England		Elevated serum creatinine found in both exposed groups. Urinary N-acetylglucosaminidase activity, a marker of proximal tubular damage, was abnormal in the unprotected group
Al-Qaoud TM, 2011 (290)		Cross-sectional		5533 participants		All occupations		UK		Components of the metabolic syndrome explain the relation between low SES and low eGFR
Ragnaud JM, 1994 (150)		Retrospective		30 cases of leptospirosis		High risk occupations		France		67% of acute renal failure
Schillinger F, 1999 (151)		Retrospective		6 cases of leptospirosis		High risk occupations		France		6 cases of acute renal failure, two cases associated with occupational exposure
Jacob S, 2007 (291)		Retrospective		269 patients with glomerulonephritis diagnosed between 1994 and 2001		Solvent exposure: toluene/xylene, gasoline, fuel and gas-oil and ketones		France		18% exposed to solvents. High risk of progression to ESRD among machinery fitters and assemblers, plumbers/welders, and those handling printing inks and petroleum products
Strady C, 2009 (152)		Retrospective		99 cases		Farmers		France		Leptospirosis-associated nephropathy
Miailhe AF, 2019 (153)		Retrospective		160 patients		Occupations that have contact with animals		France		63% of hepatorenal injury. Leptospirosis associated with specific occupations
Scheer V, 2020 (292)		Case report		One competitive runner		A 110-km trail race in mountainous terrain		France		Severe acute kidney injury
Gentile G, 2021 (154)		Retrospective from 2004 to 2018		88 cases of leptospirosis		Leptospirosis in army where chemoprophylaxis with doxycycline was not applied		France		Higher incidence in Martinique and Mayotte and 40% of severe renal forms
Nuti M, 1993 (293)		Retrospective		817 cases of positive serology to hantavirus and leptospirosis		265 forestry workers, 82 rangers, 395 farmers, and 75 hunters		Italy		Renal failure
Bek M, 1996 (155)		Case report		One man with leptospirosis		Sewage drain worker		Germany		Acute renal failure and death
Kreuzer M, 2021 (294)		Retrospective		35204 former employees		Former employees of a former Uranium Ore Mine		Saxony and Thuringia, Germany		Mortality from lung cancer was higher than general population but there was no increase death from renal disease
Olszyna DP, 1998 (156)		Retrospective		1986-1990: 229 1991-1995: 159		Occupational leptospirosis		Netherlands		Decrease in cases over 10 years. Weil’s syndrome in 10%
Kolwijck E, 2011 (157)		Case report		Farm worker		Occupational leptospirosis		Netherlands		Acute renal failure
Adjei DN, 2017 (295)		Cross-sectional analysis		The HELIUS study of 21,433 adults of multiethnic origin (Surinamese, Ghanaians, Turks, Moroccans and Dutch)		Low occupational level		Netherlands		Low occupational level in the ethnic minority groups associated with CKD
Spinder N, 2021 (296)		Case-control study		601 cases born with urinary tract malformations and 5602 controls		Maternal occupational exposure		Netherlands		Association between maternal organic solvent exposure and CAKUT
Brattebø G, 1991 (297)		Case report				Off-shore fisheries		Norway		Crush injury and AKI
Rönsholt FF, 2009 (158)		Case report		56-year-old man		Working in henhouse		Denmark		Leptospirosis-associated kidney failure
Martin-Reina J, 2021 (298)		Cross-sectional		39 women (18-45 years)		Women farmers exposed to pesticides		Spain		Subclinical kidney damage (elevated urinary KIM-1)
Wrońska-Nofer T, 2015 (299)		Case-control		53 male foundrymen and 40 male controls		Steel plant workers		Poland		Renal scintigraphy demonstrated kidney function alterations that correlated with lead exposure
Canney M, 2018 (300)		Cross-sectional analysis		4,996 participants from The Irish Longitudinal Study on Ageing		Disadvantaged childhood socioeconomic position (based on father’s occupation)		Ireland		Low childhood socioeconomic position was strongly associated with CKD in women

In the USA, a great interest grew in the Mesoamerican experience with the aim to prevent CKD in US agriculture population (301). Several studies in agricultural workers of Hispanic origin and residing in California and Florida confirmed the recurrent AKI due to heat stress and dehydration during the work shift (265, 271–273, 276, 277). Smith et al. assessed 50 patients, undocumented workers (coming from Mexico), who received frequent emergent-hemodialysis and found out that they had high-risk occupations with heat stress and exposure to pesticides and lead paint fumes in construction (97). A large study in the USA of 23,692 citizens showed that lower neighborhood SES was associated with higher risk of CKD (278).

In Japan and South Korea, the occupational kidney disease was studied from another perspective. Tsurugano et al. from Japan showed an association between job stress, high blood pressure and kidney disease in 1231 male office workers (281). In 2020, an analysis of 20,851 workers from South Korea revealed an association between long working hours and decreased eGFR (284). Kim et al. in South Korea showed that occupational noise in females is associated with high CKD prevalence and this could be related to the increase in the sympathetic nervous system activity (285). In Taiwan, taxi drivers have higher albuminuria and metabolic syndrome (286). Another interesting review from high-income countries is about the role of newer halogenated hydrocarbons and their risk of DNA injury and altered renal function. Anesthesiologists are at risk of chronic exposure if air-conditioning systems are not protective and the best solution would be to use intravenous anesthetics (302).

## Recommendations

### Altitude

After reviewing the impact of altitude on kidney health we can suggest the following measures:

-Healthy patients should climb to high altitude on several days to allow the acclimatization or acclimation process and to avoid acute mountain sickness and a severe decline in glomerular filtration rate.
-Hypertensive patients should monitor their blood pressure at high altitude.
-Patients with pre-established CKD should be aware of the risk of pulmonary edema; guidelines do not recommend a different hemoglobin target than those at sea-level. In case of acute mountain sickness, CKD patients should be aware of an adjustment of dosage of medications prescribed.
-Transplant patients do not need to change their immunosuppressant medications’ dose at high altitude.
-Living at high altitude may predispose to albuminuria and hypertension and renin-angiotensin inhibitors are the preferred drugs in these cases.
-People working in the space should be aware of the side effects of acute hypobaric hypoxia and avoid abrupt exposure to low atmospheric pressure.

### Seasonal weather variation and extreme temperatures

Although seasonal variation is not extensively studied, climate change is expected to lead to an increase in extreme temperatures. Consequently, the following points are important to highlight:

-Hypertensive patients should be aware of the risk of exacerbation of hypertension during extreme cold weather.
-Vulnerable populations like elderly and children should be protected and well hydrated during heat waves to avoid acute kidney injury.
-Lupus erythematosus patients should be aware of the seasonal variation of renal flares and the impact of humidity, cold and ultraviolet radiation.

### Occupation

Despite all the evidence behind occupational renal risk, screening for occupational kidney disease is still not universally recommended. Several challenges may hinder screening and should be considered (303). One should assess first if reduction of exposure is possible, second if the evidence of using one biomarker for screening reflects well a preclinical stage of kidney disease, third if diagnostics are available in certain places, fourth if workers would accept screening, finally it is important to figure out whether screening will be followed by an existing effective intervention.

-In highly endemic regions or communities, screening is important and applying preventive interventions is crucial (304–306).
-Working in heat -whether outdoor in agriculture or construction or even as athletes, or indoor in kitchens or foundries-, should be recognized as an occupational kidney hazard and preventive interventions should be applied: hydration should be emphasized (307), it should include water and electrolytes and should be sugar-free; rest periods are very important as well as proper access to healthcare and ensuring as much as possible shade in outdoor activities and proper air conditioning in indoor jobs to avoid maintaining the dehydration and kidney injury.
-Gender disparities among different occupational kidney diseases need to be further evaluated in the future. Reports showed higher prevalence of agriculture-related kidney disease in men and occupational noise-related kidney disease in women. However, two recent reviews by García et al. and Swartling et al. found important sex differences in the detection and prevalence of kidney disease with lower diagnosis of chronic kidney disease and lower SES in women globally (308, 309).
-Despite the long history of heavy metals and pesticides, examples from the most vulnerable groups in the world show that there is insufficient awareness and vigilance about their nephrotoxicity. There is inconsistent coordination between governments, researchers, decision makers to prevent the high-risk exposure of agricultural and industrial workers and to mitigate the increase in kidney disease cases.
-It goes without saying that health is as important as productivity.

### Need for coordination

The experience from Sri Lanka and Mesoamerica showed the challenges that emerge when efforts are fragmented, the decision-makers are not committed and the different stakeholders are not well coordinating (310, 311). Involvement of international experts, mobilizing funds for research and preventive interventions are also needed. Occupational Safety and Health has a major role in preventing kidney disease worldwide with a need to update guidelines such as adding electrolytes to water during hydration, such as putting kidney health on the bigger agenda. Occupational Safety and Health can also ensure trainings in workplaces that are at high risk of exposure to nephrotoxic hazards (312).

### Research

Evidence-based research is very important to build relevant health policies and implement effective and efficient interventions. However, challenges are numerous when conducting research in vulnerable populations.

We can learn from the challenges of researchers in Mesoamerica. Three themes were hindering the progress of research (311). The first one is the influence of government and industries’ interests, the second is the scarce human and financial resources and third the pragmatic challenges of undergoing research projects.

Researchers from North America also faced challenges when conducting occupational studies in Mesoamerica (313). The lessons learned are: clear definition of roles and responsibilities and of mutual expectations, good discussion of research outcomes, setting realistic goals and timelines, active listening especially in a multilingual environment, sharing personal experience with humility, not presuming that expertise entails trust, creating a sense of community.

Finally, standardization of terms and research protocols is crucial when focusing on a hot health topic. In this present review, several publications on occupational kidney disease were missed because authors did not mention the term “occupation” or “occupational” in their abstract or keywords. As an example of standardized protocol, “The Disadvantaged Populations eGFR Epidemiology (DEGREE) Study” protocol was published in 2017 (314). The DEGREE project aims to characterize the distribution of eGFR in multiple LMICs in a standardized way that helps make international comparisons. This protocol has been a model implemented and still being implemented in several countries.

## Conclusions

As a summary, environmental factors, socioeconomic status and occupation are all independent risk factors for kidney disease but they often act synergistically. Living in extreme temperatures and low socioeconomic neighborhood, even in high-income countries, is adding on the burden of global kidney disease. These weather and occupational conditions are widening the chasm in kidney health between the wealthy and disadvantaged populations. Altitude and cold-related kidney disease are also two topics that require more research and evidence especially with the climate change and the risk of extreme temperatures. Seasonality has important impact on the practice of nephrology and hypertension management. And most of all, the workplace should ensure safety to our kidney health and international efforts from scientists, ministries of health, nephrology societies should be gathered to address with standard policies and protocols the old and newly identified hazards that may jeopardize the kidney’s well-being.

## Author contributions

MA contributed to the conceptualization and literature search. MA contributed to the first draft. DC revised the manuscript. Both authors approved the final version.

## Conflict of interest

The authors declare that the research was conducted in the absence of any commercial or financial relationships that could be construed as a potential conflict of interest.

## Publisher’s Note

All claims expressed in this article are solely those of the authors and do not necessarily represent those of their affiliated organizations, or those of the publisher, the editors and the reviewers. Any product that may be evaluated in this article, or claim that may be made by its manufacturer, is not guaranteed or endorsed by the publisher.
